# Development of a Personalised Device for Systemic Magnetic Drug Targeting to Brain Tumours

**DOI:** 10.7150/ntno.76559

**Published:** 2023-01-01

**Authors:** Priya Patel, Areej Alghamdi, Gary Shaw, Christopher Legge, Maggie Glover, Danielle Freeman, Harry Hodgetts, Erica Wilson, Faith Howard, Sarah Staniland, Aneurin J Kennerley, Duncan Wood, Robert Moorehead, Charlotte Hadfield, Ola Rominiyi, Jon Griffin, Spencer J Collis, Sam Hyde, Marcus Crossley, Martyn Paley, Munitta Muthana

**Affiliations:** 1 Faculty of Medicine, Dentistry and Health, University of Sheffield, Sheffield, S10 1RX, United Kingdom.; 2 Oncology and Clinical Research, University of Leeds, Leeds, LS2 9JT, United Kingdom.; 3 Department of Chemistry, University of Sheffield, Brook Hill, Sheffield S3 7HF, United Kingdom.; 4 Department of Computer Science, University of York, York, YO10 5GH, United Kingdom.; 5 Medical AMRC, University of Sheffield, Factory of the Future, Catcliffe, Rotherham, S60 5TZ, United Kingdom.

**Keywords:** Magnetic Nano Particles, Brain Tumours, Magnetic Drug Targeting, Murine Models

## Abstract

Delivering therapies to deeply seated brain tumours (BT) is a major clinical challenge. Magnetic drug targeting (MDT) could overcome this by rapidly transporting magnetised drugs directly into BT. We have developed a magnetic device for application in murine BT models using an array of neodymium magnets with a combined strength of 0.7T. In a closed fluidic system, the magnetic device trapped magnetic nanoparticles (MNP) up to distances of 0.8cm. In mice, the magnetic device guided intravenously administered MNP (<50nm) from the circulation into the brain where they localised within mouse BT. Furthermore, MDT of magnetised Temozolomide (TMZ^mag+^) significantly reduced tumour growth and extended mouse survival to 48 days compared to the other treatment groups. Using the same principles, we built a proof of principle scalable magnetic device for human use with a strength of 1.1T. This magnetic device demonstrated trapping of MNP undergoing flow at distances up to 5cm. MDT using our magnetic device provides an opportunity for targeted delivery of magnetised drugs to human BT.

## Introduction

Magnetic drug targeting (MDT) is a promising method of concentrating magnetic nanoparticles (MNP) such as iron oxide at a target site *in vivo* 1-5. The pioneering idea proposed by Freeman et al (1960), suggested MNP can be transported through the vascular system and concentrated to a particular point in the body with the aid of a magnetic field 6. In the late 1970s, researchers proposed the use of MDT to deliver anti-cancer drugs to specific sites in the body 7-9. In addition, attaching the drugs to MNP has been shown to not only reduce the drug dose but also the cytotoxic effects to healthy tissues and organs 2, 9-12, enhancing the MDT technology. There have only been a small number of clinical trials (phase I/II) using MDT attached with anti-cancer drugs such as doxorubicin performed 13. Lubbe *et al* found the application of a magnetic field close to the tumour resulted in successful targeting of magnetically labelled epirubicin in 50% of patients and was well tolerated. Organ toxicity was not seen to increase but high drug doses (>50mg/m^2^) were required which resulted in epirubicin toxicity 13. Similarly, Koda et al. 14 demonstrated MDT with magnetised epirubicin was well tolerated in the 14 patients studied when magnetic fields (0.5T) were applied directly to the tumours for 60-120 min. In this study, the distance between the magnet and tumour was less than 0.5 cm and successful targeting was observed in 6/14 patients. Furthermore, successful delivery of magnetised doxorubicin via the hepatic artery was demonstrated in patients with hepatocellular carcinoma 14. These MDT studies are promising; however, one must consider the applicability of this approach in tumours present in deeper parts of the body, e.g., the brain, where direct drug injection is not possible and where the external magnet is not strong enough to penetrate the site of interest. Indeed, the effective range of currently available electro and permanent magnets limit such a technique to tissues close to the body surface (<20 mm) 15-18.

To overcome this, researchers have attempted to exploit the magnetic field gradient coils inherent to all magnetic resonance imaging (MRI) systems, to steer ferromagnetic particles (or cells containing them) to a target site 19. Early studies in pigs demonstrated this concept by steering a 1.5mm ball bearing 5 cm inside the right carotid artery of the animal using the gradient coil currents of a standard 1.5 T MRI system 20, 21. MRI could also be used to steer iron-labelled human peripheral blood mononuclear cells in a vascular model 22. We also magnetically labelled macrophages armed with an oncolytic virus and demonstrated a reduction in tumour size and improved mouse survival using MRI to guide this cell-based therapy in primary and secondary murine prostate cancer 23. MR guided ultrasound has also been used for the enhancement of doxorubicin uptake in prostrate 24 and BT 25 in rodent models. Although these have shown success in targeting therapies towards tumour sites, in reality, the use of MRI scanners for this treatment strategy could be cumbersome. MRI scanners are known to have a high per patient use cost, are in high demand resulting in long patient waiting times and many patients experience claustrophobia and anxiety during their examination, with some patients requiring sedation before their scans 26. In light of this, we wanted to develop a small, portable device using magnets that would allow for the 'guidance' of magnetised therapeutic agents to inaccessible targets (>5 cm), with the aim to provide an alternative strategy to MRI and help to avoid the discomfort and associated costs.

In the first instance, we sought to target murine BT. Malignant glioma is one of the most common and aggressive BTs in humans and the most common type of BT representing 30% of all central nervous system tumours (CNST) 27. Glioma patients have a median survival time of approximately 1 year, and only 5% survive more than 5 years 28. Chemotherapy is one of the most important strategies for glioma treatment. However, the impermeability of the Blood-Brain Barrier (BBB) limits options for glioma chemotherapy, and there are only a few BBB-permeable chemotherapies (e.g., temozolomide, carmustine) clinically available 26, 29. The strictly limited selection of drugs and resistance to these chemotherapies 30 imposes a great challenge and, therefore, the development of magnetically guided strategies that enable drugs to penetrate the BBB are urgently needed.

Herein, we report the design and development of a magnetic device for targeting anti-cancer drugs to BT when delivered via circulation. We magnetised temozolomide (TMZ^ mag+^) with silica coated Fe_3_O_4_ MNP; which are well documented for ease of preparation and handling, availability, affordability, biosafety and the possibility of both imaging and targeting the drug(s) of interest to the desired location 31. MDT of TMZ^mag+^ resulted in improved tumour targeting, tumour shrinkage and extended mouse survival. We then scaled up our MDT device setting a central axis to generate a magnetic field that can focus to greater distances (>5 cm) than existing magnets and has the potential to target tumours in inaccessible locations in humans.

## Materials and Methods

### Magnet design using FEMM

The magnetic device was designed using Finite Element Method Magnetics (FEMM). FEMM is a finite element solver for low frequency magnetics in the 2D plane and provides a way to model the magnetic field strength, measured in units of the Tesla (T). In addition, a scripting language (Lua 4.0) is also integrated with the program so that simulations of MNP underneath the magnetic field strength could be observed. The aim of the configuration created in FEMM was to use the minimal number of magnets to achieve the maximum field and field gradient at a given depth away from the magnet surface. The configuration created used five single (3mm × 3mm × 3mm) magnets with fields pointing along different axes and the magnetic strength was maximised along the central plane of the magnet device.

### Creating the magnetic device

The configuration designed using FEMM was replicated using rare earth Neodymium Iron Boron (Nd-Fe-B)-grade N42 strength magnets (First4magnets.com). The size of each magnet was 3mm × 3mm × 3mm and five individual magnets were glued (Loctite Control Super Glue Adhesive) together to create the device. To determine how the magnetic field changed with distance and the force generated a Gaussmeter (GM 08, HIRST, Magnetic instruments Ltd) was used to measure the magnetic strength in T.

### Testing the magnetic device in a closed fluidic flow system

*In vitro* experiments were devised to test the ability of the MD to 'trap' Fe_3_O_4_ MNP (<50nm, Sigma-Aldrich) in water (5mg/ml) across a range of distances. The MNP were pumped in an open phantom flow model using a peristaltic pump with an infusion rate of 10ml/min (Master Dual Pump, WPI). The MD was positioned at three different distances (0 cm, 0.4 cm and 0.8 cm) above the tubing. These distances were comparable to the typical depth of tumours in a mouse 32. To quantify the amount of MNP 'trapped' by the MD, the residual MNP left within the tube were measured using Inductively Coupled Plasma Spectroscopy (ICP).

### Inductively coupled plasma Atomic Emission Spectrometer (ICP-AES)

A Spectro-Ciros-Vision Inductively Coupled Plasma Atomic Emission Spectrometer (ICP-AES) was utilized to calculate the quantity of Fe as a ratio of metal ions for the MNP used throughout this project. Different MNP concentrations were dissolved in Aqua Regia (King's water), a mixture of concentrated nitric and hydrochloric acids (HCL) at a molar ratio of 1:1 of nitric acid and HCL. 1ml of sample was added to 1ml of Aqua Regia solution and diluted in 8ml of DH_2_O. The samples were kept for 24 hours before ICP. Fe content (mg/l) was determined from atomic absorption 33.

### Digestion of tissues

Post-mortem tissue samples (e.g., brain, lungs, spleen, liver) were digested using ICP-AES for Fe quantification. Samples were dried prior to digestion. The dried sample mass was calculated using the recorded mass of the sample before and after drying.

The dried sample mass was between 16 and 400 mg, so add 6 ml of concentrated nitric acid followed by 2 ml of concentrated perchloric acid. The tubes are transferred to the hot block and the temperature increased up to 150 °C. Then the temperature is gradually ramped up to 200 °C (takes ~1 hour). This gradually boils off the nitric acid and should leave a colourless/pale yellow solution.

### Manufacture of MNP

The following materials were needed to create MNP; Ferric chloride hexahydrate (FeCl_3_.6H_2_O) (Fe (III)), ferrous chloride tetrahydrate (FeCl_2_.4H_2_O) (Fe (II)), tetraethylorthosilicate (TEOS), (3-aminopropyl) triethoxysilane (APTES) which were all purchased from Merck. Temozolomide (TMZ) purchased from Sigma Aldrich (100mg) was conjugated to the MNP.

### Synthesis of spherical Fe_3_O_4_ core shell MNP

Fe_3_O_4_ MNP were synthesized in accordance with^34^. Fe (II) chloride (1.0 mmol) and Fe (III) chloride (2.0 mmol) were dissolved in 45 mL deionised water followed by 3 ml of aqueous ammonia (25%) and stirred for 1 hour under N_2_ continuous flow. The following synthesis of creating the core-shell of the MNP was adapted from Liu et al. 34. This was achieved by dissolving Fe (II) chloride in 45 ml of 2.0ml TEOS which was added to the suspension of MNP in deionised water and stirred at 1500 rpm for 2 hrs at room temperature. For the introduction of the amine functional groups on the surface of MNP, 1.0g of MNP was dispersed in 50 ml of ethanol in a three-necked flask and placed in an ultrasonic bath for 10 minutes so the particles could disperse. Following this, 1.0 ml of 3-Aminopropyl triethoxysilane 99% (Sigma-Aldrich) (APTES) was added to the suspension and this mixture was stirred mechanically at 60 °C under N_2_ flow for 6 hrs. The synthesised MNP were separated using an external magnet and washed with deionized water and ethanol several times and then dried under vacuum at 50 °C for 24 hrs.

### Chemotherapy conjugation to functionalised MNP

Conjugation of MNP with TMZ (creating TMZ^mag+^) was accomplished by a reaction between the amine group present on the functionalised MNP and the carbonyl group of the drug via Schiff base chemistry. The amino-functionalised MNP (100 mg) were dispersed in 15 ml of ethanol and 2 drops of glacial acetic acid (acting as a catalyst) were added. TMZ (10 mg) was added to the resulting colloidal dispersion and the reaction was carried out at room temperature for 48 hrs, therefore leading to the chemical conjugation of TMZ to the MNP via imine linkage (also referred to as a Schiff base reaction). The TMZ-conjugated core-shell MNP were separated using a magnetic separator and washed several times with deionized water and ethanol and dried under vacuum at 50 °C for 24 hrs.

### *In vitro* characterisation of functionalised MNP

#### X-ray diffraction

The MNP were dispersed in deionized water and dried under vacuum at 50 °C for 24 hrs leaving a dry powder. Powder XRD patterns were collected using a standard X- ray diffractometer with Bragg-Brentano geometry (Bruker AXS- Advance) with a sealed CuK radiation (*λ=* 1.5418 Å). The Diffraction pattern was measured in the 2θ range from 20-80°.

#### FT-IR

Fourier-Transform- Infrared Spectroscopy (FT-IR) was used to show the silica formation on the surface of the Fe_3_O_4_ MNP and the presence of TMZ. MNP were ground with Potassium Bromide (KBr) then pressed into a disc and the spectrum recorded from 4000-400cm^-1^ with a resolution of 4cm^-1^ on an Elmer Perkins Spectrum Two.

#### TEM

Transmission Electron Microscopy (TEM) was used to visualise and assess the diameter of the MNP and was carried out using a FEI Tecnai T12 Spirit TEM with a Gatan Orius 1000b 11 Megapixel bottom mounted camera linked to Gatan Digital Micrograph software. A water - cooled charger coupled device (CCD) camera captured images of MNP. MNP were dispersed in water and deposited on carbon-coated copper grids.

#### UV-VIS

Drug loading was determined by measuring the absorbance of MNP in ethanol and acetic acid solutions using a UV-vis spectrometer (Agilent Technologies, Santa Clara, CA).

#### Magnetometry

A vibrating sample magnetometer (MPMS-3 VSM-SQUID) was used to measure the magnetisation as a function of magnetic field strength (Oe) up to 36.6 °C (309.75 Kelvins). The hysteresis cycle was measured with a maximum field of 10 KOe.

### Cell culture

Human peripheral blood mononuclear cells (PBMC) were isolated buffy coats obtained from the Sheffield Blood Transfusion Service as previously published 35. Briefly, the PBMC layer was collected following centrifugation over Ficoll (Sigma Aldrich, UK) and cultured overnight in IMDM and 2% human AB serum (Sigma Aldrich, UK). The murine CT-2A - luciferase labelled cells were a gift from Dr Mihaela Lorger (University of Leeds) and the human brain tumour cell lines (U138 and T98G) were purchased from the ATCC. These are established brain tumour cell lines that closely resemble human disease. The murine CT-2A luciferase-labelled cells were kept in a temperature-controlled incubator in T75 tissue culture flasks (NUNC, UK) and cultured with DMEM (Lonza, UK), supplemented with 2 mmol1^-1^ L- glutamine, 100 U ml^-1^ penicillin and Foetal bovine serum (FBS, Biosera). Cell lines were routinely mycoplasma tested and genotyped by PCR.

### Cell-based assays

Alamar Blue assays were used to assess cell viability following incubation with dilutions of MNP (5 mg, 2.5 mg, 1.25 mg, 0.625 mg, 0.3125 mg). MNPs, MNP+Silica and TMZ were added to cells (CT-2A, U138 & T98G) seeded on 96 well plates at a density of 5×10^3^ cells per well. After 24 hrs Alamar blue was for 4 hrs and viability was assessed using a spectrophotometer (SpectraMax M5^e^). Cell viability was also determined using TOPRO3 stain (Invitrogen). All FACS data was analysed on an LSRII flow cytometer (BD Biosciences) using Flowjo software (Tree star).

### Intracranial surgery

Male C57Bl/6 mice (8-12 weeks) were obtained from Charles River, UK. All mouse procedures were conducted in accordance with the UK Home Office Regulations under the Animals (Scientific Procedures) Act 1986 and the Home Office Project License (PPL: 70/8670, the ARRIVE (Animal Research: Reporting of *In vivo* Experiments) guidelines and the University of Sheffield Animal Welfare Ethical Review Body (AWERB). The University of Sheffield Animal Welfare and Ethical Review Body approved all the *in-vivo* experiments used in this study. All animals were kept in ventilated cages with food and water provided ad libitum. To avoid observer bias, cages were re-labelled by another person in our animal unit before recording measurements, and the recordings were scored blind to the treatment. The CT-2A tumour cell line 36 was developed specifically for ganglioside distribution in murine neural tumours 37, 38. All animals underwent intracranial surgery and were randomised. CT-2A (1×10^6^) cells were placed in 10 µl DMEM and kept on ice. Mice were first anaesthetised using gaseous isoflurane (2.5% isoflurane in 100% oxygen at 3.5L min^-1^) within a closed anaesthetic chamber. Mice were transferred to the stereotaxic frame kindly provided by Dr Jason Berwick, University of Sheffield (Kopf instruments). The surface was cleaned with hibiscrub then the skull exposed. The position of the bregma was identified and using this as a reference the position of the injection was determined using a Vernier scale. We wanted to inject the CT-2A cells (2×10^5^/2µl DMEM) into the right-hand side cortex using the following coordinates medial- lateral (x): 2mm, anterior- posterior (y): + 2mm. Once this position was marked, a 1mm burr hole was made using a dental micro- drill (0.8 mm) stopping at dura. A 5 µl- Hamilton syringe (Model 65 RN with a manual plunger, SLS) fitted with a glass capillary needle (30-gauge, small hub, 2 inch, point style: 4, SLS) with a tip diameter of 60-80 μm (inner and outer diameter of the tip) was inserted (vertically) 1 mm from the surface per minute. Once the needle was at 4 mm depth (z), the needle was withdrawn 1 mm to create a pocket (needle is at depth 3 mm) that the CT-2A cells (2×10^5^/2µl DMEM) can be injected into. The cells were injected 1 µl every 1 minute and once the required 2 µl was injected, the needle is kept in position for 2 minutes. After which, the needle was slowly withdrawn again 1 mm every minute.

### Designing the helmet

A design for a helmet was needed so that the mice could move around freely with the magnetic device in place on top of the head. A cast of a mouse's head was made using silicon. From this, a 3D printed helmet that could fit on top of the mouse's head and could encase the magnetic device was created. Mice were acclimated to wearing the helmet short periods of time prior to the start of the experiments.

### Tumour growth

Tumour size and growth was monitored by bioluminescence imaging using the IVIS Lumina II imaging system (Caliper Life Sciences). The mice were injected intraperitoneally with 90mg kg^-1^ D-luciferin (Caliper Life Sciences) dissolved in sterile water and anaesthetized using 2.5% isoflurane (Abbott Scandinavia AB) in 100% oxygen at 3.5L min^-1^ (for induction) in the anaesthesia chamber connected to the imaging system. Images were taken every 3 minutes as a sequence of 10 images for every group of mice, once a week. Automatic contour regions of interest were created, and the tumour sizes or tumour radiance were quantified as photons per second per square centimetre per steradian. Progression and spread of tumours were evaluated by calculating the tumour radiance values from inoculated mice in each group. Tumour- bearing mice were used in experiments approximately 5-7 days following implantation and studies were allowed to progress until tumours reached maximum permitted size (~2.0 × 10^7^ photons/second) so that survival studies were possible.

### MRI to visualise MNP particles in-vivo

Mice were imaged using MRI 7T small bore magnet with a 660mTm^-1^ gradient insert, Bruker BioSpec AVANCE II, 310-mm bore, MRI system B/C 70/30. The mice were first anaesthetized using an induction chamber with continuous isoflurane (2.5% isoflurane in 100% oxygen at 3.5L min^1^). Once anaesthetised, mice were injected intravenously with MNP (5 mg/kg in 100 ul of PBS) either with or without the magnet placed on top of the head for 30 minutes. After 30 minutes the magnetic device was removed, and mice were placed within the MRI bore to be scanned with T2 and T2* weighted images. The control group received intravenous PBS (100 ul) as well as the placement of the magnetic device for 30 mins. Mice were culled immediately after the imaging took place where tissues and organs were removed for post-mortem analysis.

### Targeting MNP in a murine model of glioma

In experiments where TMZ therapy was delivered, mice received the following intra-venously: Vehicle (0.1% DMSO, 0.9%PBS), TMZ (0.5mg) + Vehicle (0.1% DMSO, 0.9%PBS), TMZ^mag+^+Vehicle (5mg/kg - 0.5mg + 0.1% DMSO, 0.9%PBS) and TMZ^mag+^+Vehicle+ magnetic device (5mg/kg + 0.5mg +0.1% DMSO, 0.9% PBS, 30 minutes). Tumour growth was monitored over time using bioluminescence imaging (3 times/week) as described above. Survival studies were performed, and tumours were allowed to grow to a maximum of ~2.0 x 10^7^ photons/second or until animals displayed symptoms of poor health (or loss of 20% body weight). Kaplan-Meier estimates were used to generate survival curves.

### Tissue Analysis

For post-mortem analysis, organs (brain, lung, liver and spleen) were removed from mice and preserved in 10% formalin for 48 hrs. These were then transferred to phosphate-buffered saline (PBS) and tissue processed to wax, after which they were wax embedded into blocks. Sectioning was done at 5µm thickness using Leica Semi-Automated Rotary Microtome (Model RM2245).

### Immunohistochemical analysis

Tissue sections were stained with H&E, Perl Prussian Blue (20% Hydrochloric Acid, 10% Potassium Ferrocyanide) and counterstained with nuclear fast red solution for improved contrast and MNP detection 39. CD31 (1:100; AbD Serotec) was used to visualise the tumour vasculature and peroxidase activity was localised with diaminobenzidine (Vectastain Elite ABC kit, Vector Labs). Slides were visualized using the Hamamatsu NanoZoomer XR (Hamamatsu, Hertfordshire, UK). The area of necrosis within the whole-tumour section was determined visually, and the proportion of necrotic nonviable tumour areas over the whole section was calculated using ImageJ (National Institute of Health). For each group, the mean percentage of necrosis and standard error were calculated. The results are presented as mean tumour necrosis (%) of all tumours (five slices per each tumour) in each treatment group.

### Magnetic device scale up with stronger targeting capabilities

FEMM was used to model a magnetic device using Neodymium Iron Boron (NdFeB)-grade N52 magnets, 1cm^3^ (Magnet Expert, UK) at different magnetisation orientations. The device was assembled based on the FEMM models (as above). The magnet was assembled by Magnet Expert, UK. This was necessary as it was impossible to hold the magnets due to repulsive forces from the individual magnets which required specialist equipment.

### Phantom models

A 3D phantom flow model was devised to assess MNP 'trapping' of our scaled up magnetic device suitable for human use. This consisted of a 3D printed head (Advanced Manufacturing Research Centre (AMRC), University of Sheffield, UK) based on optical and MR scan data, which geometrically mimics the surface of the head of a patient. Tumours were printed from synthetic polymer, 3 cm in diameter (typical of a human brain tumour) with a complex network on the inside and an inlet and outlet for connecting to a flow system (Masterflex L/S. Series Peristaltic Pumps) at an infusion rate of 150ml/min to mimic the blood flow in human vessels. The tumour was designed so that it could be placed at different distances (0, 5 and 10cm) underneath the 3D printed head (skull thickness of 0.8 cm) to mimic the potential locations of the tumour within the brain. The trapped MNP were visualized using a dedicated 3T neonatal MRI (GE Healthcare, Schenectady, NY, USA). Once the targeting was complete, both the inlet and outlet of the 3D tumour phantom models were sealed with a silicone sealant to prevent the particles from leaking out and keep them inside the tumour. The tumours were scanned using a dual gradient echo sequence (TE= 4.60ms, 20ms) to measure T2*. This was achieved by drawing a contour over the tumour on one image and copying this to the other echo. The mean value of both regions of interest (ROIs) were taken to perform the calculation 40. The signal intensity ratios (SIRs)-pixel values were extracted from the same region of interest from each tumour for scans with two different echo times i.e., TE 4.60 ms and TE 20 ms. This was applied for each tumour with the different MNP concentrations. The following equation was applied to calculate T2*:



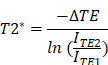



Where 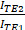 is SIRs of TE_2_ 20 ms and TE_1_ 4.60 ms respectively.

### Statistical analysis

Data results are presented as mean ± s.e.m or SD (Prism 7; GraphPad Software). A two-tailed Student's t-test was used to analyse the statistical significance of the data unless otherwise stated. Differences were deemed significant with a p value of <0.05.

## Results

### Design and development of a magnetic drug-targeting device using FEMM

**Figure 1:**
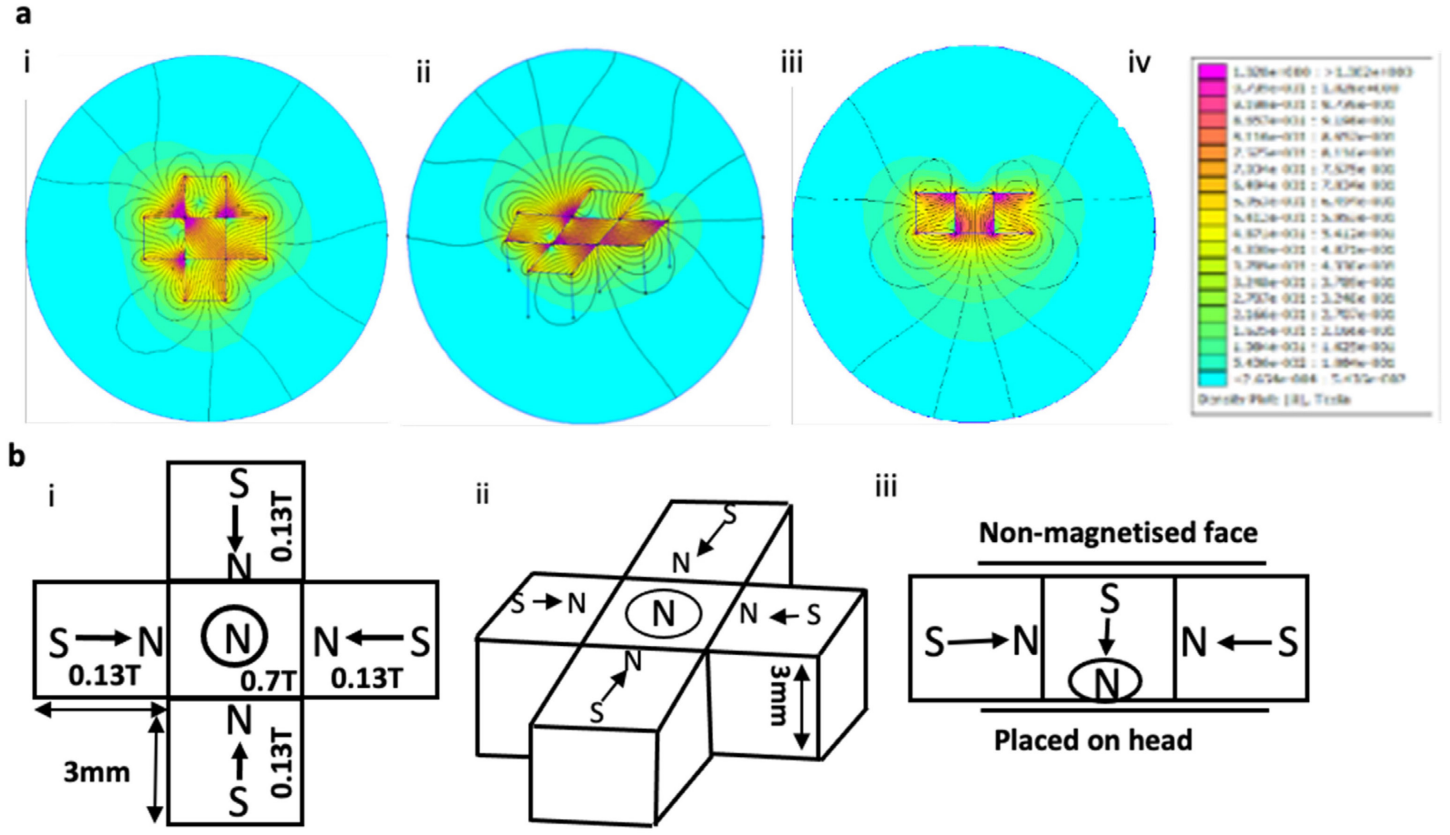
** Magnet design and configuration using FEMM**. **a**) Images from FEMM software shows the shape and number of magnets used in the magnetic device; i) front face, ii) side view, iii) cross section, iv density plot bar shows the intensity (measured in 'T') of the magnetic field strength. **b**) Schematic drawing of the magnetic device showing the direction of the magnetic polarities (S=south, N=north). I) Front facing view with magnetic polarities and magnetic field strength showed in 'T' for each magnet, ii side view showing same magnetic polarities and dimensions, iii) cross section with magnetic polarities.

The design of the magnetic device was performed using software called FEMM. This software solves magnetic problems in a 2D plane. The FEMM modelling software evaluated a variety of designs and the final configuration has an increased magnetic field strength directed towards the centre magnet (Figure 1ai, aii, aiii). Within FEMM, all the north facing magnets have been directed inwards all facing each other, with the centre magnet perpendicular to the surrounding magnets (Figure 1bi, bii, biii). The magnetic device was designed with the following parameters: 1. A magnetic field strength strong enough to penetrate through a mouse's brain (~1 cm depth). 2. Lightweight to allow for free movement whilst wearing the device. 3. Sized aptly so it can fit comfortably on the top part of a mouse's head (Supplementary Figure 1). Using a Gaussmeter the magnetic field strength of the central magnet was found to be strongest (0.7T) compared to the peripheral magnets (~0.13T) (Figure 1bi).

### Magnetic device 'targets' MNP undergoing continuous flow

**Figure 2:**
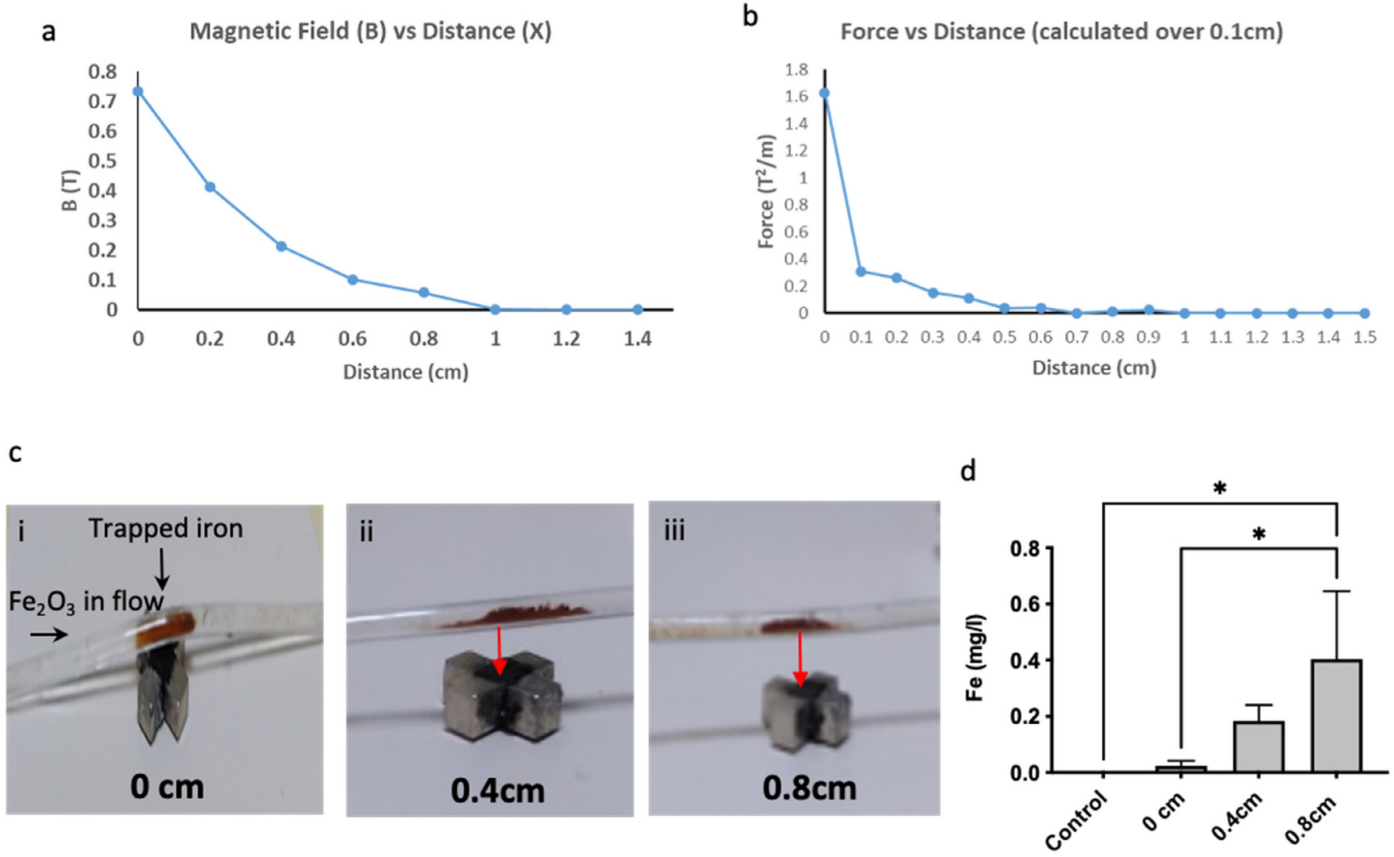
** MNPs were trapped whilst undergoing flow using the magnetic device. a**) Magnetic field was measured as a function of distance (steps of 0.2cm). **b**) Magnetic trapping force of the magnetic device was measured by calculating the product of the magnetic field and magnetic field gradient at different distances. **c**) Shows the magnetic device at three different distances; i - 0cm, ii - 0.4cm, iii - 0.8cm. 5mg of MNP was mixed in water and flowed through the tubing using a peristaltic pump within a closed loop system. **d**) Analysis using ICP shows the amount of MNP collected at the end of the tubing with the magnetic device in place where control samples contain no iron. Data are mean ± SEM and analysed using a one-way ANOVA with Tukey's multiple comparison test, *p > 0.05.

Following the construction of the magnetic device, tests to determine the magnetic field gradient and force changing with distance was performed. The local field gradient measured in B(T) (Figure 2a) and force measured in T^2^/m (Figure 2b) as a function of distance. The force was calculated from the product of the magnetic field and local field gradient at each measured location. This showed that as the distance increased, the field gradient and force was seen to decrease as expected (Figure 2a, 2b). The magnetic ability to 'trap' these silica-coated MNP at a range of different distances was also assessed using our magnetic device. To test if the magnetic device would be able to target MNP across the depth of the mouse brain, we took measurements and found an approximate depth of ~1 cm (supplementary figure 1). This distance was also modelled within FEMM (Supplementary Figure 2) to show the decrease in magnetic field strength at three distances (0cm, 0.4cm, 0.8cm). In addition, to test 'trapping' at these distances, MNP undergoing flow were successfully trapped at 0cm (where the magnet is in direct contact with the plastic tubing (flow rate - 10ml/min) (Figure 2ci), at 0.4cm (Figure 2cii) and 0.8cm (Figure 2ciii). As expected, less MNP (Fe mg/ml) were trapped as the distance increased (Figure 2d). Despite this, the forces generated by the magnetic device provide the potential to guide MNP across the full depth of a mouse's brain.

Next, we prepared silica coated MNP. These were typically 20-35 nm diameter, with a negative zeta potential and retained their magnetic susceptibility (Table 1). We incubated silica coated MNP with human and murine brain tumour cell lines and compared toxicity to naked MNP. At high concentrations (2.5-5 mg/ml), silica-coated MNP induced cell death in the human glioblastoma T98G cells (Supplementary Figure 3) although this was not evident in the other cell lines. We also determined how peripheral blood mononuclear cells (PBMC) responded to MNP as these cells are encountered in circulation. At concentrations of less than 5 mg/ml, the silica coated MNP were found to be least toxic in these cells (Supplementary Figure 4).

### Magnetic targeting increased the amount of iron delivered to the brain

**Figure 3:**
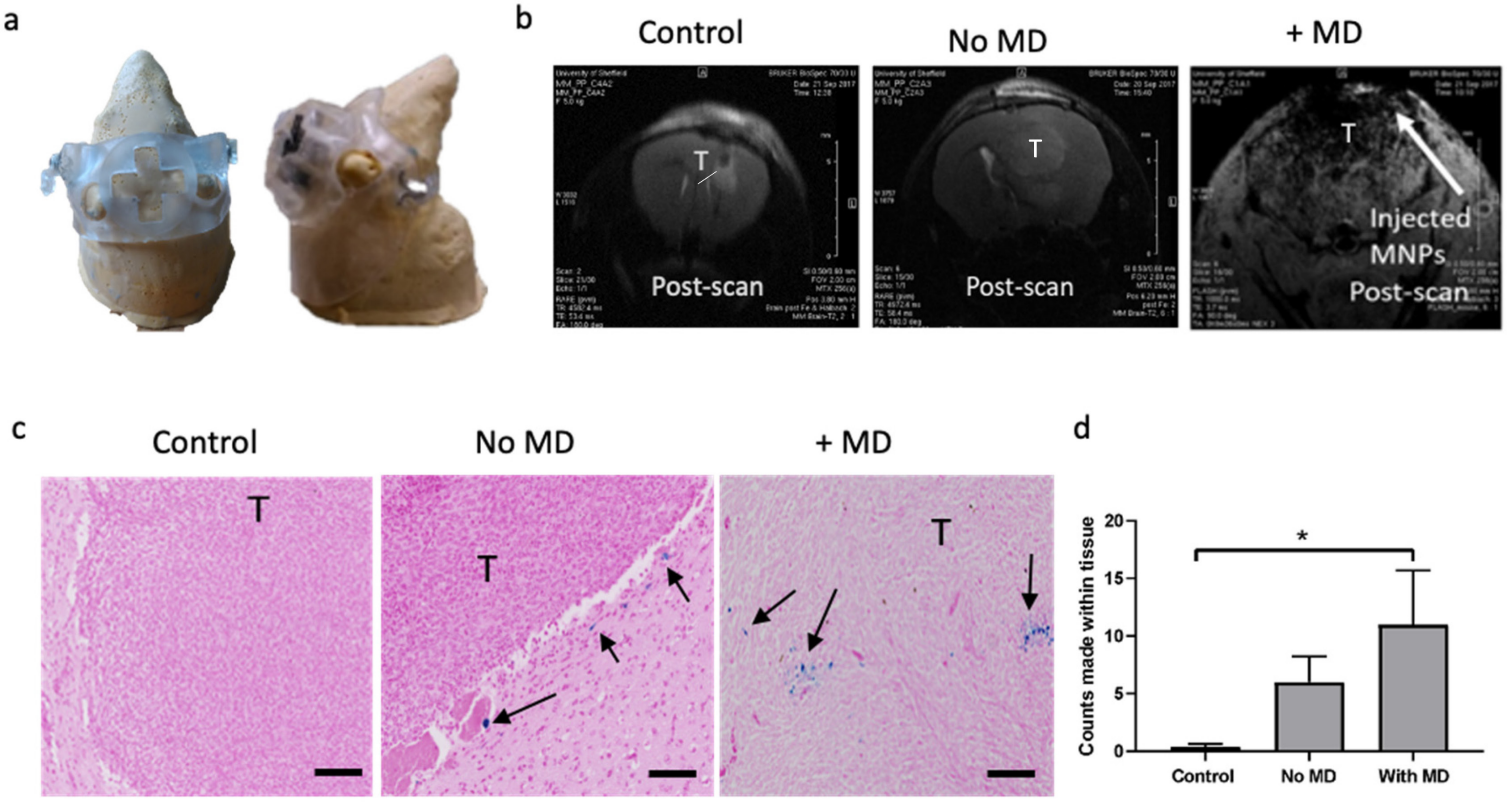
** The magnetic device increases the amount of iron to the brain. a**) A 3D printed helmet was made to fit around a murine model made from clay. The helmet also housed the magnetic device. **b**) MRI (7T) scans showing coronal sections of murine brains and tumour (T and highlighted in black) 30 minutes after i.v. injection of either PBS (control), MNP with MD (+MD) and without MD (No MD). **c**) Representative images of Prussian Blue staining within the tumour area for each treatment group is shown (scale bar=50um). Arrows show the presence of iron (in blue) in the periphery and inside the tumour tissue. **d**) Manual counts of positive Prussian Blue staining throughout the whole tissue section, n=4. Data are mean ± SEM and analysed using a one-way ANOVA and with Tukey's multiple comparison test, *p < 0.05.

Given that the magnetic device was able to 'trap' MNP in fluid flow *in vitro*, next we assessed if this could attract MNP administered into circulation across the BBB, into the brain. Mice were first acclimatised to wearing the 3D printed helmet (Figure 3a) that housed the magnetic device for short periods of time (30 minutes daily for 5 days). Mice were then injected with either PBS (Control), MNP with the magnetic device (+MD) and without a magnetic device (no MD) (Figure 3b). Mice were then monitored by MRI for 40min -1 hour and as shown in Figure 3b the accumulation of MNP was more prominent in the presence of the magnetic device compared to the other groups. This was further demonstrated by measuring the iron content using ICP-ACES on post-mortem brains of MNP (5 mg/kg) in the presence of the magnetic device (Supplementary Figure 5a). This was also confirmed in the post-mortem histological analysis of the tissues, where staining for MNP using Prussian Blue revealed a significant increase in the accumulation of iron within the brain and predominantly within the tumours where the magnetic device was placed (Figure 3c & 3d). ICP analysis also demonstrated an increase of iron content in the brain following magnetic guidance (Supplementary Figure 5a). Despite the accumulation of MNP in the brain, post-mortem analysis revealed that magnetic guidance did not affect the overall brain tumour vascularity (CD31 positive staining) and necrosis (Supplementary Figure 5a, b, c) also no statistical significance was found between the groups (although, there is a trend towards the increase in vessels in the group with magnetic biodistribution). Biodistribution of the MNP was also evaluated in other organs 41 and as expected MNP particles were detected in the liver, lung and spleen of the mice after 30 minutes of magnetic guidance and this resulted in a trend towards less detectable MNP in these organs (Supplementary Figure 6).

### Chemical synthesis of MNP conjugated to anti-cancer drug Temozolomide (TMZ)

Next, we magnetised TMZ, to treat gliomas for use with our magnetic device. First, we confirmed susceptibility of two human BT cell lines (T98G, U138) and one murine BT cell line (CT-2A) to TMZ. As expected, the results showed that after 24 hours of exposure increasing concentrations of the chemotherapy led to reduced cell viability, indicating the ability of the individual agents to kill all these cell lines, respectively (Supplementary Figure 7a-c).

**Figure 4:**
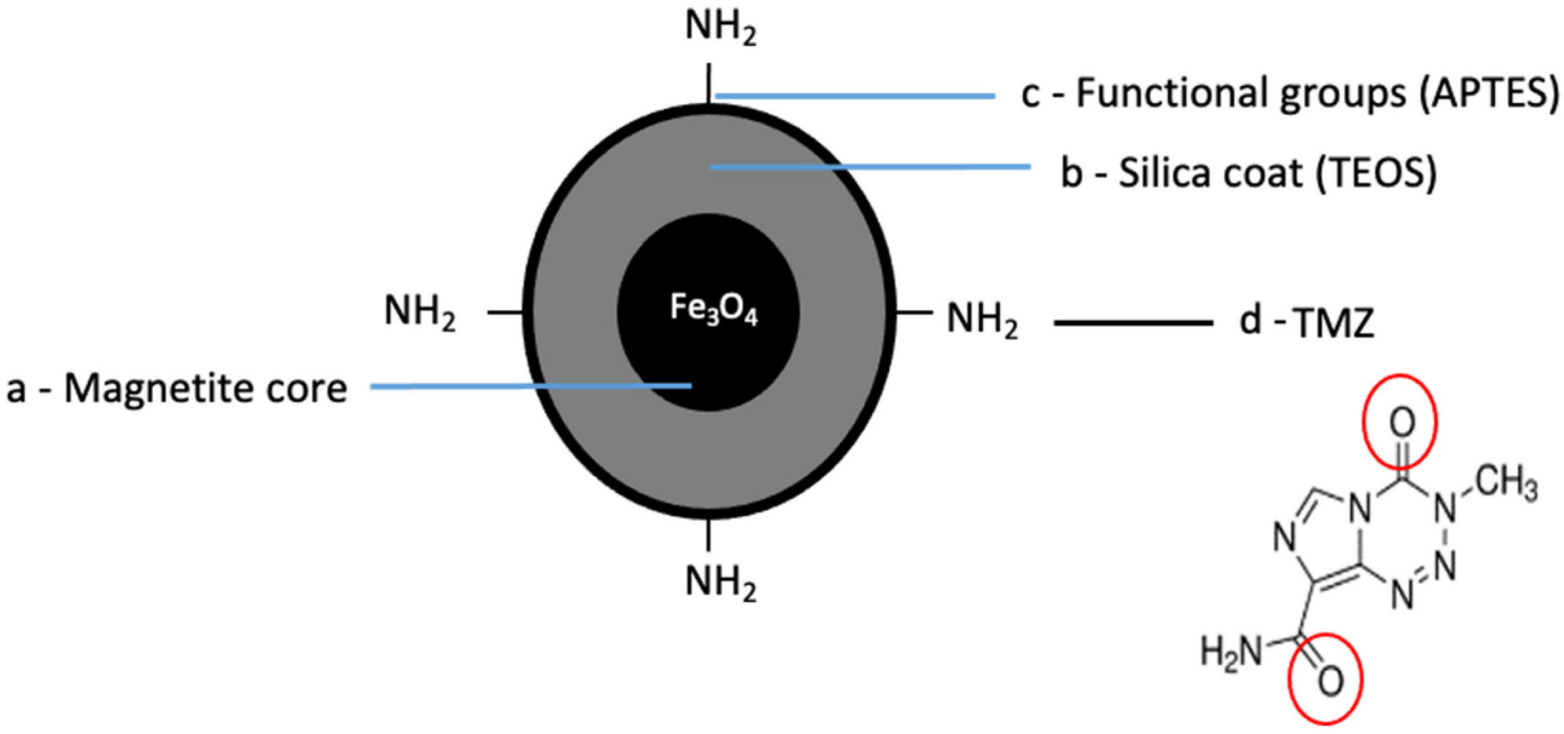
** Schematic of chemical synthesis of MNP conjugated to anti-cancer drug. a**) The core is made up of magnetite (Fe_3_O_4_), **b**) MNP were coated with a silica- based shell covering the surface (TEOS), **c**) The synthesized MNP were modified by APTES providing amine functional groups (-NH_2_) created on the surface of silica coated Fe_3_O_4_ MNP, **d**) TMZ was separately attached to the functionalised silica coated Fe_3_O_4_ MNP through Schiff base condensation.

We prepared the TMZ^mag+^ using a Schiff base condensation technique. This involved creating a condensation reaction between a carbonyl-containing derivative with primary amine groups in the presence of metal ions (Figure 4a-d).

### Characterisation of MNP conjugated to anti-cancer drug Temozolomide (TMZ)

**Figure 5:**
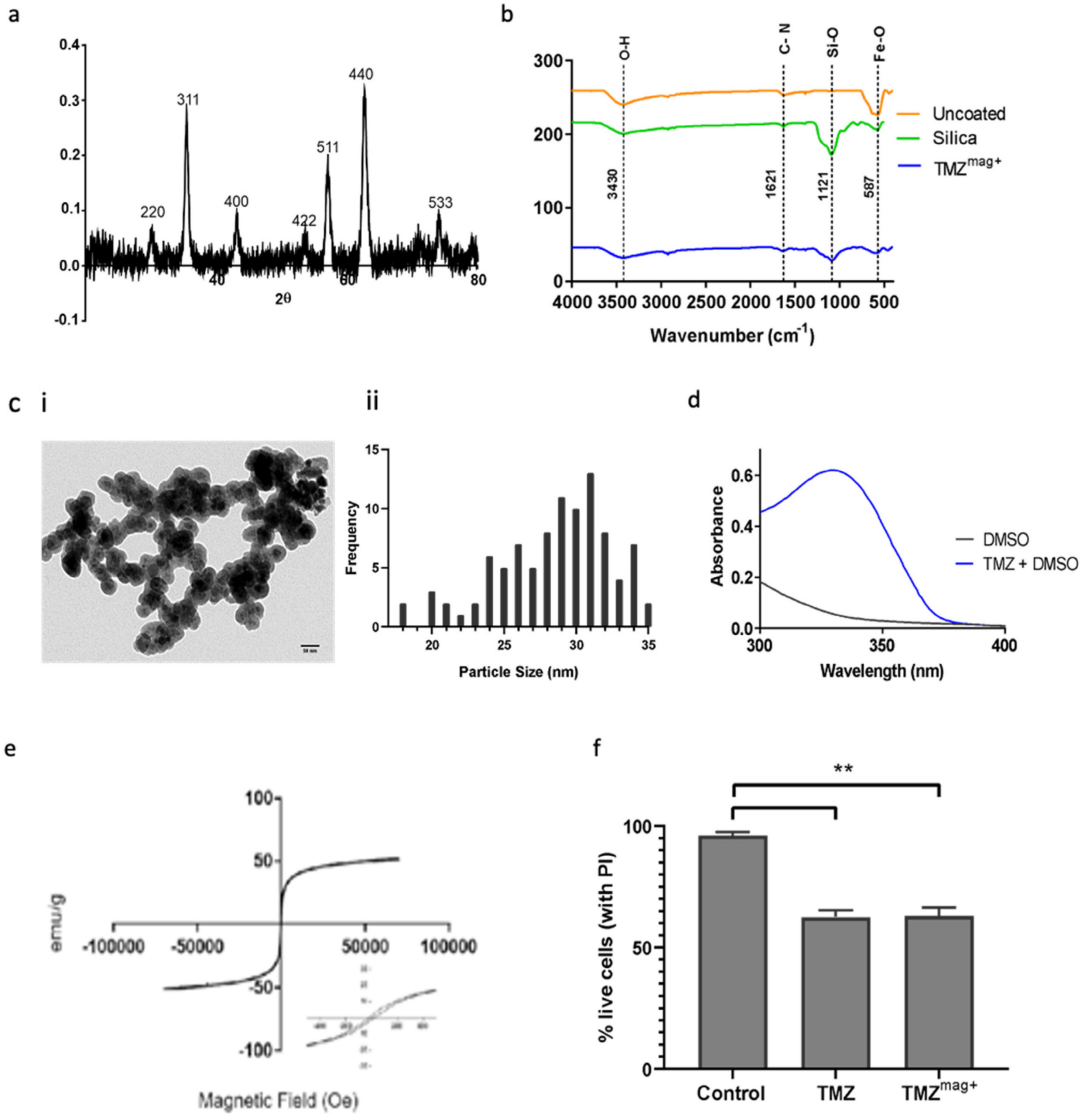
** Characterisation of TMZ^mag+^. a**) X-ray diffraction patterns shows evidence of the crystalline structures of naked MNP, **b**) FT-IR shows the presence of the crystalline structures in the uncoated, silica coated MNP and silica coated TMZ^mag+^, **c**)** i** Representative TEM image shows the silica coated Fe_3_O_4,_ and **ii** average diameter measured using Image J measuring tool, scale = 50nm, **d**) UV-VIS absorption spectra shows the presence of TMZ^mag+^ 24 hours the complex was made, **e**) Magnetisation curve of TMZ^mag+^ measured at body temperature (36.6 °C, 309.75 K), **f**) TMZ^mag+^ effectively kills brain tumour cells (CT-2A) *in vitro*. Cells (1x10^6^) were incubated with Vehicle (Control), TMZ (0.5mg) and TMZ^mag+^ for 24hrs. Propidium iodide was added to cells to assess cell viability/death by flow cytometry using the LSRII. Data shown are the mean ± SD of N=3 independent experiments and analysed using one-way ANOVA where *p*=0.05.

X-ray diffraction (XRD) revealed characteristic peaks which correspond to (220), (311), (400), (422), (511), (440) and (533) Bragg reflections for the MNP in the TMZ^mag+^ (Figure 5a). The reflections observed in the XRD patterns can be assigned to MNP crystal structure. FT-IR shows the surface properties of the MNP (Figure 5b). The characteristic peak of a Si-O bond appeared as a relatively broad band at 1121cm^-1^ assigned to the corresponding stretchable vibration demonstrating the presence of silica on the surface of MNP 38. The band at 587cm^-1^ shows the presence of Fe-O and two bands at 1621 cm^-1^ and 3430 cm^-1^ are the C-N and O-H bonds, respectively (Figure 5b) 38. TEM shows the MNP (black) and the surrounding silica (grey) which encapsulates the MNP within the size range of (20-35 nm) diameter with an average diameter of 28.5 nm (Figure 5c). UV-VIS absorption spectra (absorption at 328 nm) was used to confirm the presence of TMZ^mag+^ 24 hours later (Figure 5d). This suggests that the TMZ had not degraded. The magnetic properties were further evaluated by VSM; Figure 5e shows the magnetisation curves of the MNP and confirms that TMZ^mag+^ exhibited paramagnetic properties. TMZ^mag+^ was tested for its ability to kill murine BT cells *in vitro* and both TMZ alone and in the magnetised complex (TMZ^mag+^) were equally effective at killing tumour cells (Figure 5f).

### Magnetic drug targeting of TMZ^mag+^ slowed brain tumour growth

**Figure 6:**
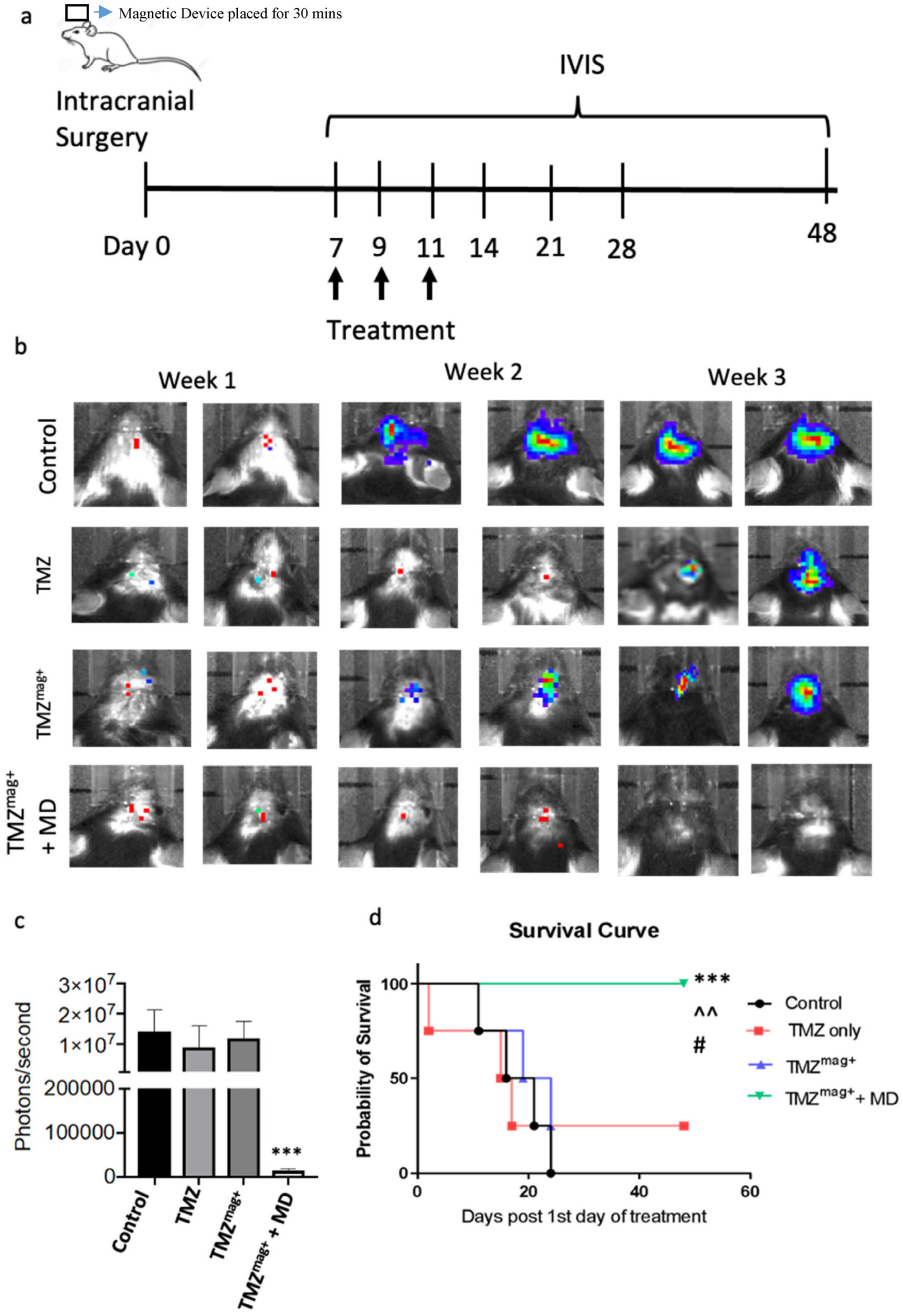
** Magnetic guidance of TMZ inhibits brain tumour growth**. Mice underwent intracranial surgery followed by injection of luciferin labelled CT-2A brain tumour cells into the cortical region. Tumour growth was tracked by IVIS imaging. **a**) Schematic of the treatment strategy. Mice received 3 doses of intravenous injection consisting of 100μl of either PBS only, TMZ, TMZ^mag+^ with and without a magnetic device (MD) (placed for 30 mins) on day 7 following intracranial surgery and successful BT progression, **b**) Representative IVIS images taken twice a week for three weeks after the treatment in groups, **c**) Regions of interest were created around the tumour area to measure bioluminescence in photons/second on day 21** d**) Survival data of mice up to day 48 when the experiment was terminated (TMZ^ mag+^ + MD), the median survival for the control and TMZ^mag+^ was 18 days; TMZ^ mag+^ only group was 24 days. A total of N=4 mice per group. A chi-square test of independence was performed to examine the relationship between the survival rates compared to the number of days post-surgery within the groups (x^2^ (1) = 3.636; p = 0.01). ***p=0.001 TMZ^mag+^ vs PBS, ^*p*=0.01 vs TMZ and #*p*=0.05 vs TMZ^mag+^ (No MD).

The targeting and therapeutic efficacy of TMZ^mag+^ was tested using the magnetic device in the CT-2A mouse glioma model. This model is established and accurately represents numerous GBM characteristics, including the intra-tumoral cellular heterogeneity and the proliferative and metabolic profiles, these are suitable for testing novel therapies 38. A schematic of the treatment schedule is shown in Figure 6a. The treatment included i.v. injections of 100 ul of either Vehicle (Control), TMZ, TMZ^mag+^, with and without the magnetic device (placed for 30 mins only). The concentration of silica-coated MNP in the drug was 5 mg/ml and TMZ 0.5 mg/ml, these were selected based on the earlier toxicity studies and published protocols 30. As expected, tumour growth was reduced in the presence of TMZ and TMZ^mag+^ for up to 2 weeks post treatment compared to the vehicle treated mice, whereas in the group where magnetic guidance of the chemotherapy was applied, tumour growth was further inhibited (Figure 6b). The cumulative size of the tumours within each of the four experimental groups was determined by applying a region of interest over the tumour and tumour radiance (photons/second) was calculated at week 3-post treatment. This revealed significantly smaller tumours in the TMZ^mag+^ compared to the other groups where tumours continued to grow over time and by week 3 were of similar size to the controls (Figure 6c). We did see an effect with TMZ; however, this was not seen to extend mouse survival beyond controls because tumours were seen to regrow (approximately after the second week). Furthermore, all mice in the magnetic guidance group survived significantly longer and until the experiment was terminated on day 48 (Figure 6d). We performed further post-mortem analysis of the brains taken from the mice at week 3 post-treatment. No MNP were detected in the brains of the mice by the end of the experiment (Supplementary Figure 8a-d) and interestingly, BT were visibly reduced in the magnetic guidance group (Supplementary Figure 8d). On a similar note, no MNP were detected in the livers, lungs and spleens (Supplementary Figure 9) in any of the treatment groups by the end of the experiment. This is reassuring as it suggests that the iron is eliminated thus preventing the potential of any unwanted toxic effects.

### Scaled up magnetic device with stronger targeting capabilities

**Figure 7:**
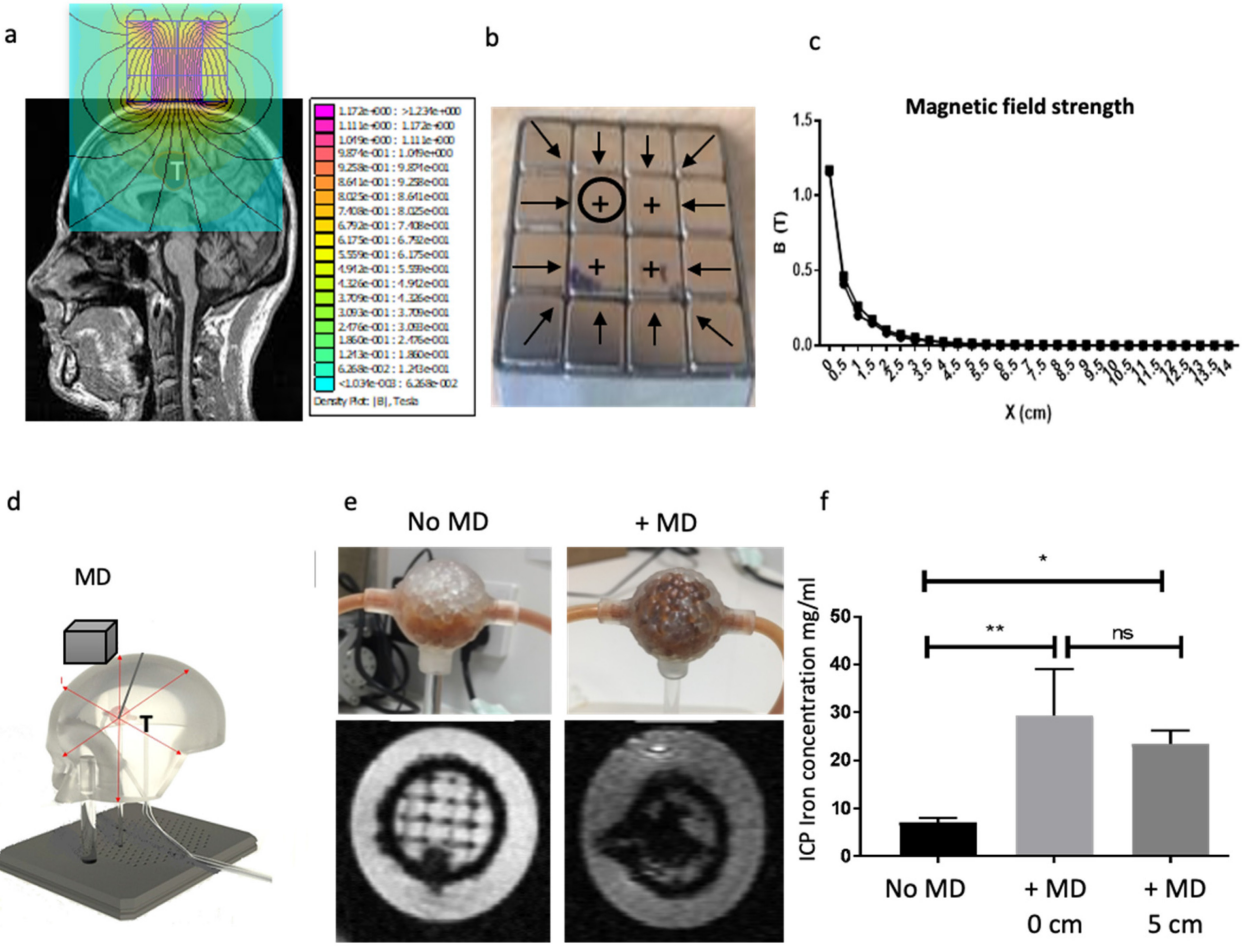
** Scalable magnetic device with strong MNP targeting distances. a)** 2D simulation model of a magnetic device based on Neodymium-iron-boron (FeNdB-52) magnets was designed using MRI scans from glioma patients in FEMM. **b**) The FEMM model was assembled. Black circles in the magnet refer to the position where the magnetic field strength measurements were taken, perpendicular to the magnet's surface. The sign (+) on the magnets refers to the magnetisation orientation set with θ=90°. The black arrows display the magnetisation orientation set with θ=180°, which are orthogonally magnetised towards the centre as well as 45° from the corners. Each magnet element had a volume of 1 cm^3^. The assembled device was encased in steel. **c**) Magnetic field strength measured at the surface of the magnet with and without the steel case over distance **d**) A phantom flow model (skull thickness - 0.8cm) was developed to test 'trapping' of MNP targeting (MD=magnetic device, T = tumour). **e**) Using this model, 5 mg/ml MNP were pumped in an open loop through a flow system using a syringe pump at a rate of 10 ml/min. The MD was placed at 5 cm away from the head. Once the trapping of MNP was complete, both the inlet and outlet of the tumours were sealed to prevent the particles from leaking out and scanned using MRI. Representative T2* weighted MR images shows MNP trapped within the tumours in the presence of the MD. The scans were obtained using a 3T neonatal MRI system with a dual gradient echo sequence, TE=4.60ms and 20ms. **f**) MNP were flushed out from the tumours and measured using ICP. The tumours were located at two different distances in the phantom model: 0 cm and 5 cm away from the magnet. Data are the mean and SD of N=3 experiments. All data was analysed using a one-way ANOVA *p <0.01 and **p =0.001.

Here, we developed a scalable magnetic device suitable for humans. We used scanned MRI images of human BT as a reference for approximate tumour positions and size (Figure 7a). Several factors were considered in the magnetic device design including the size, strength of each magnet and the orientation of magnetisation. Lua scripting modelling of MNP showed that smaller MNPs require less magnetic force to move them towards the target site (Supplementary Figure 10). This result resonates with observations made by others 42 where it was shown that the amount of magnetic force required to move MNP to the target site is directly proportional to the volume of the particles. In addition, the use of small MNP has the advantage of reduced aggregation and subsequent clotting inside blood vessels 43. In our study, we used MNP in the size range of 25-35 nm, these were also silica-coated to prevent further aggregation. Figure 7b shows the orientation of the magnetisations. FEMM modelling showed that multiple layers of magnets resulted in a stronger magnetic field strength, gradient, and force at the centre of the magnetic device (Supplementary Figure 11a). The field gradient and magnetic force were then calculated in FEMM based on the magnetic field strength measurements (Supplementary Figure 11b). The field strength was measured over a range of distances in FEMM for each layer and showed that combined 3 layers had stronger field strength (Supplementary Figure 11c). Next, we assembled the magnetic device in 1, 2 and 3 layers (Supplementary Figure 11d). To validate the FEMM simulations, measurements of the magnetic field strength, force and gradient were taken for each layer using a Gaussmeter (Supplementary Figure 11e). The maximum magnetic field generated by the first layer was 0.93T at the surface of the magnet. This value increased as the number of layers of magnet increased to 1.06T and 1.08T for the second and third layers respectively (Supplementary Figure 11e). Next, we optimised the magnetic device further by filling in the corners with additional magnets and embedded this within a steel case, together this helped concentrate the magnetic field and reduce escape from the corners of the model (Figure 7b). This also increased the magnetic field strength from 1.08T to 1.17T (Figure 7c). To assess if these depths will be applicable in MDT in humans, we attempted to simulate this *in vitro* in a simplified 3D phantom flow model (Figure 7d). The magnetic device was placed directly on top of the 3D printed head and the tumours were placed underneath the head at different distances away from the magnetic device whilst the MNP underwent fluid flow for 5 minutes. MRI data showed MNP trapped within the tumour in the presence (and absence) of the magnetic device (Figure 7e). ICP also confirmed at distances 0 and 5cm the magnetic device was equally effective at targeting MNP within the tumour (Figure 7f). T2* was seen to decrease as the concentration of MNP increased (Supplementary Figure 12a). As expected, increasing the concentration of MNP resulted in increased MNP targeting whilst increasing the distance of the magnetic device from tumour decreased the targeting efficacy (Supplementary Figure 12b).

## Discussion

Here we demonstrated the feasibility of MDT with an external magnetic device designed to penetrate the BBB for use in applications where the targeting depth is a few centimetres and particularly suitable for treating diseases that are accessible to within ∼5 cm of the magnet surface. In the first instance, we designed a miniature magnetic device for use in mice consisting of a custom-made design whereby the magnetic field is concentrated to the centre of the device and can penetrate through the full depth of a mouse brain. We demonstrated this effectively guided MNP from circulation into murine gliomas. In addition, MNP were found to be more concentrated in the tumour region and not in the surrounding area under the influence of the magnetic device, this may result from enhanced permeability of a leaky tumour vasculature to increase the therapeutic retention 44. Next, we used the magnetic device to 'guide' magnetised temozolomide (TMZ^mag+^) towards developing BT and observed that the tumours developed at a slower rate than the control group and the TMZ treated mice in the absence of the magnetic field, with enhanced mouse survival post treatment. The mice were continually monitored for signs of adverse reactions (e.g., weight loss, reduction in movement). Already a small number of studies in murine brain tumour models have demonstrated the feasibility of MDT following intravenous delivery of MNP in rodent glioma models, where MNP were seen to accumulate in the interstitial space within tumour tissue in the presence of a magnetic field 10, 45. Typically, these studies used a single disk magnet so were restricted to tumours near the surface due to a rapid decrease of magnetic field strength as the distance of the tumour increases. Because of this, permanent magnets are only useful for superficial tumours. For MDT to become a reality there is a great need to generate stronger and deeper magnetic fields and field gradients (penetrating to more than 20 mm) from external magnets 17.

A recent study simulated how magnets can be clinically translatable to human size. They modelled a U-shaped permanent magnet design with a potential to target MNP (100 nm) up to a depth of 5-7cm 5 and with a minimum force of 3T^2^/m. Using a different approach, the same team have recently used a novel MRI-guided ablation technique able to perform thermoablation within subcutaneous tumours 46. Barnsley et al., presented a number of optimised light-weight designs for permanent magnetic devices based on the Halbach array, with a force of about 0.3 T^2^/m at 5 cm distance from the magnet 47, 48. In their experiments, they noted that layered cubic elements were more effective at targeting and trapping magnetically functionalised microbubbles against flow at distances of 20 mm 40. With this in mind, we have for the first time designed, developed and tested a scalable magnetic device based on the layered Halbach array, which can generate a minimum force of 20.7 T^2^/m and achievable targeting depths of up to 5 cm. Such a device can be used to target drugs to the brain or other body organs with similar depths such as breast, neck or legs. In general, by increasing the number of layers, the volume of the device is increased and could become inconvenient for use with patients. However, our model consists of small magnetic elements (1cm × 1cm × 1 cm) made it possible to make a small magnetic device (400g for a 3cm × 3cm × 3cm device). The other benefit of using small sized magnetic elements was that it was easy to change the individual orientations of element magnetisation to generate a stronger magnetic field. For future developments, the magnetic device could also be susceptibility matched to the head using magnetic fillers e.g., iron loaded epoxy resin, this will focus the field on the head thus preventing magnetic field leakage.

Finally, our system offers the following advantages over the use of other externally applied magnetic devices: 1) a high local magnetic field and field gradient able to penetrate distances up to 5cm 2) portable and lightweight 3) safe to handle 4) no need for an energy source 5) the design of the device can be personalised so the magnetic field and gradient can be altered to suit the tumour size and depth 6) ability to position the device over any part of the body 7) use of MDT with our device could reduce the dose of chemotherapy and prevent unwanted systemic side effects.

## Conclusion

The use of magnetism in treating brain tumours is an interesting area and we have seen the development of targeted delivery of magnetised drugs increase over the years. This type of delivery holds promise in treating invasive cancers. For gliomas, MDT can reduce drug concentrations in circulation as well as increase therapeutic index by localizing chemotherapy at the tumour site. To the best of our knowledge, this is the first study to report MDT is feasible for BT following systemic drug delivery using a specially designed magnetic device that can be scaled up for human use.

## Supplementary Material

Supplementary figures.

## Figures and Tables

**Table 1:** Characteristics of uncoated magnetite and silica coated magnetite nanoparticles as determined by TEM, DLS, XRD, Zeta potential and room temperature VSM

	TEM (nm)	DLS (nm)	Theoretical crystal size (nm)	Zeta Potential (mv)	Magnetic Coercivity (Oe)
Uncoated Particle	7.8	120.3	11.99	-22.9	21
Silica coated particles	20.4	81.3	16.93	-25.4	20

## References

[1] Arruebo M, Fernández-Pacheco R, Ibarra MR, Santamaría J (2007). Magnetic nanoparticles for drug delivery. Nano today.

[2] Jurgons R, Seliger C, Hilpert A, Trahms L, Odenbach S, Alexiou C (2006). Drug loaded magnetic nanoparticles for cancer therapy. Journal of Physics: Condensed Matter.

[3] Meyers PH, Cronic F, Nice CM Jr (1963). Experimental Approach in the Use and Magnetic Control of Metallic Iron Particles in the Lymphatic and Vascular System of Dogs as a Contrast and Isotopic Agent. Am J Roentgenol Radium Ther Nucl Med.

[4] Mohseni M, Connell JJ, Payne C, Patrick PS, Baker R, Yu Y (2020). Scalable magnet geometries enhance tumour targeting of magnetic nano-carriers. Materials & Design.

[5] Pankhurst QA, Connolly J, Jones S, Dobson J (2003). Applications of magnetic nanoparticles in biomedicine. Journal of physics D: Applied physics.

[6] Freeman M, Arrott A, Watson J (1960). Magnetism in medicine. Journal of Applied Physics.

[7] Senyei A, Widder K, Czerlinski G (1978). Magnetic guidance of drug-carrying microspheres. Journal of Applied Physics.

[8] Widder K, Flouret G, Senyei A (1979). Magnetic microspheres: synthesis of a novel parenteral drug carrier. Journal of pharmaceutical sciences.

[9] Widder KJ, Morris RM, Poore GA, Howard DP, Senyei AE (1983). Selective targeting of magnetic albumin microspheres containing low-dose doxorubicin: total remission in Yoshida sarcoma-bearing rats. European Journal of Cancer and Clinical Oncology.

[10] Chertok B, Moffat BA, David AE, Yu F, Bergemann C, Ross BD (2008). Iron oxide nanoparticles as a drug delivery vehicle for MRI monitored magnetic targeting of brain tumors. Biomaterials.

[11] Kheirkhah P, Denyer S, Bhimani AD, Arnone GD, Esfahani DR, Aguilar T (2018). Magnetic Drug Targeting: A Novel Treatment for Intramedullary Spinal Cord Tumors. Sci Rep.

[12] Sun C, Fang C, Stephen Z, Veiseh O, Hansen S, Lee D Tumor-targeted drug delivery and MRI contrast enhancement by chlorotoxin-conjugated iron oxide nanoparticles. 2008.

[13] Lübbe AS, Bergemann C, Riess H, Schriever F, Reichardt P, Possinger K (1996). Clinical experiences with magnetic drug targeting: a phase I study with 4′-epidoxorubicin in 14 patients with advanced solid tumors. Cancer research.

[14] Koda JV, A (2002). Walser, E. Goodwin, Scott. A multicenter, phase I/II trial of hepatic intra-arterial delivery of doxorubicin hydrochloride adsorbed to Magnetic Targeted Carriers in patients with hepatocellular carcinoma. European Journal of Cancer.

[15] Price PM, Mahmoud WE, Al-Ghamdi AA, Bronstein LM (2018). Magnetic Drug Delivery: Where the Field Is Going. Frontiers in Chemistry.

[16] Shapiro B, Kulkarni S, Nacev A, Muro S, Stepanov PY, Weinberg IN (2015). Open challenges in magnetic drug targeting. Wiley Interdisciplinary Reviews: Nanomedicine and Nanobiotechnology.

[17] Takeda S-i, Mishima F, Fujimoto S, Izumi Y, Nishijima S (2007). Development of magnetically targeted drug delivery system using superconducting magnet. Journal of Magnetism and Magnetic Materials.

[18] Nishijima S, Mishima F, Terada T, Takeda S (2007). A study on magnetically targeted drug delivery system using superconducting magnet. Physica C: Superconductivity and its applications.

[19] Felfoul O, Becker AT, Fagogenis G, Dupont PE (2016). Simultaneous steering and imaging of magnetic particles using MRI toward delivery of therapeutics. Sci Rep.

[20] Chanu A, Martel S (2007). Real-time software platform design for in-vivo navigation of a small ferromagnetic device in a swine carotid artery using a magnetic resonance imaging system. Conf Proc IEEE Eng Med Biol Soc.

[21] Martel S, Mathieu JB, Felfoul O, Chanu A, Aboussouan E, Tamaz S (2007). Medical and technical protocol for automatic navigation of a wireless device in the carotid artery of a living swine using a standard clinical MRI system. Med Image Comput Comput Assist Interv.

[22] Riegler J, Liew A, Hynes SO, Ortega D, O'Brien T, Day RM (2013). Superparamagnetic iron oxide nanoparticle targeting of MSCs in vascular injury. Biomaterials.

[23] Muthana M, Kennerley AJ, Hughes R, Fagnano E, Richardson J, Paul M (2015). Directing cell therapy to anatomic target sites in vivo with magnetic resonance targeting. Nature communications.

[24] Chen X, Cvetkovic D, Ma CM, Chen L (2012). Quantitative study of focused ultrasound enhanced doxorubicin delivery to prostate tumor in vivo with MRI guidance. Medical physics.

[25] Treat LH, McDannold N, Vykhodtseva N, Zhang Y, Tam K, Hynynen K (2007). Targeted delivery of doxorubicin to the rat brain at therapeutic levels using MRI-guided focused ultrasound. Int J Cancer.

[26] Dewey M, Hamm B (2007). Cost effectiveness of coronary angiography and calcium scoring using CT and stress MRI for diagnosis of coronary artery disease. Eur Radiol.

[27] Alphandéry E (2018). Glioblastoma treatments: An account of recent industrial developments. Frontiers in pharmacology.

[28] Gallego O (2015). Nonsurgical treatment of recurrent glioblastoma. Curr Oncol.

[29] Wang Z, Sun H, Yakisich JS (2014). Overcoming the blood-brain barrier for chemotherapy: limitations, challenges and rising problems. Anticancer Agents Med Chem.

[30] Sadighian S, Rostamizadeh K, Hosseini-Monfared H, Hamidi M (2014). Doxorubicin-conjugated core-shell magnetite nanoparticles as dual-targeting carriers for anticancer drug delivery. Colloids and Surfaces B: Biointerfaces.

[31] Badea A, Ali-Sharief AA, Johnson GA (2007). Morphometric analysis of the C57BL/6J mouse brain. Neuroimage.

[33] Moor C, Lymberopoulou T, Dietrich VJ (2001). Determination of Heavy Metals in Soils, Sediments and Geological Materials by ICP-AES and ICP-MS. Microchimica Acta.

[34] Liu G, Wang H, Yang X, Li L (2009). Synthesis of tri-layer hybrid microspheres with magnetic core and functional polymer shell. European Polymer Journal.

[35] Muthana M, Giannoudis A, Scott SD, Fang HY, Coffelt SB, Morrow FJ (2011). Use of macrophages to target therapeutic adenovirus to human prostate tumors. Cancer Res.

[36] Seyfried TN, el-Abbadi M, Roy ML (1992). Ganglioside distribution in murine neural tumors. Mol Chem Neuropathol.

[37] Binello E, Qadeer ZA, Kothari HP, Emdad L, Germano IM (2012). Stemness of the CT-2A immunocompetent mouse brain tumor model: characterization in vitro. Journal of cancer.

[38] Martínez Murillo R, Martínez A (2007). Standardization of an orthotopic mouse brain tumor model following transplantation of CT-2A astrocytoma cells. Histology and histopathology, vol 22, nº12 (2007).

[39] Muthana M, Scott SD, Farrow N, Morrow F, Murdoch C, Grubb S (2008). A novel magnetic approach to enhance the efficacy of cell-based gene therapies. Gene therapy.

[40] Anderson LJ, Holden S, Davis B, Prescott E, Charrier CC, Bunce NH (2001). Cardiovascular T2-star (T2*) magnetic resonance for the early diagnosis of myocardial iron overload. Eur Heart J.

[42] Gassner AL, Abonnenc M, Chen HX, Morandini J, Josserand J, Rossier JS (2009). Magnetic forces produced by rectangular permanent magnets in static microsystems. Lab Chip.

[43] Barnsley LC, Carugo D, Owen J, Stride E (2015). Halbach arrays consisting of cubic elements optimised for high field gradients in magnetic drug targeting applications. Phys Med Biol.

[44] Gambarota G, Leenders W, Maass C, Wesseling P, van der Kogel B, van Tellingen O (2008). Characterisation of tumour vasculature in mouse brain by USPIO contrast-enhanced MRI. British Journal of Cancer.

[45] Chertok B, David AE, Yang VC (2011). Brain tumor targeting of magnetic nanoparticles for potential drug delivery: effect of administration route and magnetic field topography. Journal of controlled release.

[46] Baker RR, Payne C, Yu Y, Mohseni M, Connell JJ, Lin F (2022). Image-Guided Magnetic Thermoseed Navigation and Tumor Ablation Using a Magnetic Resonance Imaging System. Adv Sci (Weinh).

[47] Barnsley LC, Carugo D, Aron M, Stride E (2017). Understanding the dynamics of superparamagnetic particles under the influence of high field gradient arrays. Phys Med Biol.

[48] Barnsley LC CD, Stride E (2016). Optimized shapes of magnetic arrays for drug targeting applications. Journal of Physics D: Applied Physics.

