# Supplementary material for: Development of a Personalised Device for Systemic Magnetic Drug Targeting to Brain Tumours
Source: Nanotheranostics. 2023 Jan 1;7(1):102–16. doi: 10.7150/ntno.76559 (PMC9760367; doi:10.7150/ntno.76559)
Supplement: Supplementary file 1 — Supplementary figures. [file ntnov07p0102s1.pdf]

Figure S1

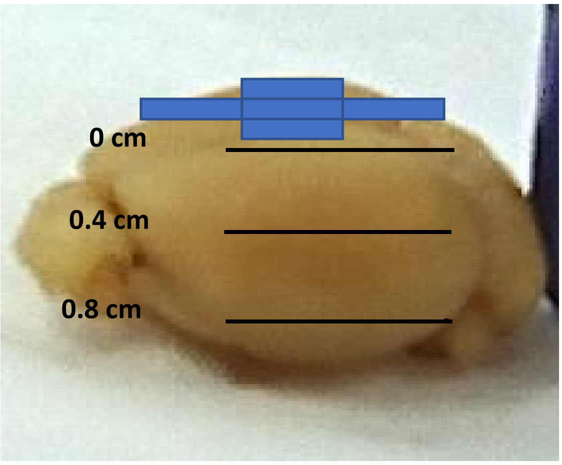

Figure S1: Image showing the approx. depth of a mouse's brain. This depth was used as a guidance for the development of the magnetic device. The depth was measured about just under 1cm. The magnetic device was intended to project the magnetic field across the majority depth of the brain, so aimed to focus on 0,0.4,0.8cm distances from the top of the surface of the brain. These distances will be used for the purpose of the in-vitro experiment. The length of the brain (~1.2cm) was used to design the size of the magnetic device (in blue). The mouse brain dimensions; length – ~1.2cm, width ~1cm, depth ~1cm and the whole brain average volume is 508.91±23.42 mm^3

Figure S2

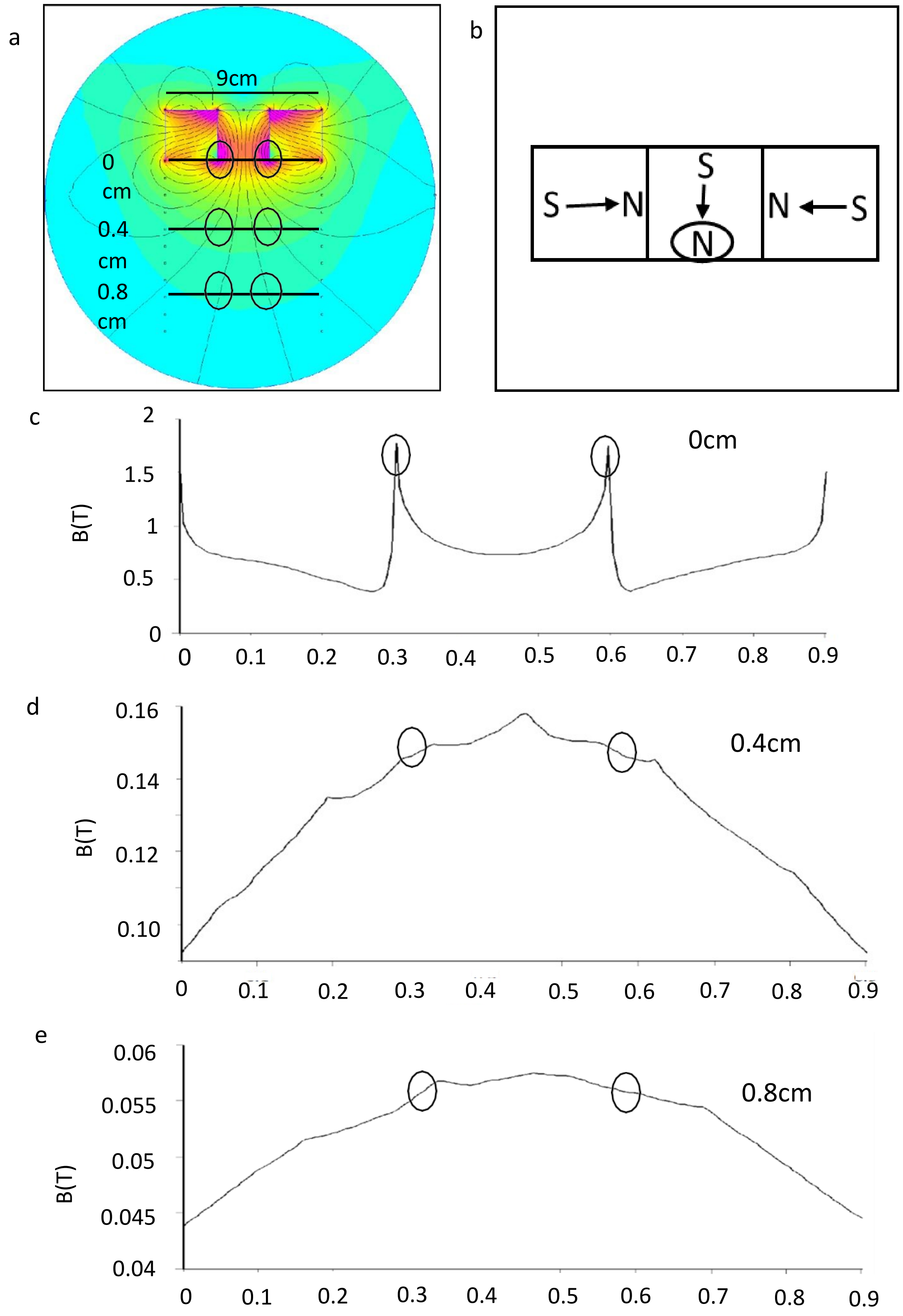
a
9cm
0
cm
0.4
cm
0.8
cm
b
S
N
S
N
N
S
c
0cm
B(T)
2
1.5
1
0.5
0
0
0.1
0.2
0.3
0.4
0.5
0.6
0.7
0.8
0.9
d
0.4cm
B(T)
0.16
0.14
0.12
0.10
0
0.1
0.2
0.3
0.4
0.5
0.6
0.7
0.8
0.9
e
0.8cm
B(T)
0.06
0.055
0.05
0.045
0.04
0
0.1
0.2
0.3
0.4
0.5
0.6
0.7
0.8
0.9

Figure S2: a) Measurements taken from FEMM to show the magnetic field strength (T), three distances from the magnetic device. **a)**- shows a cross section of the magnetic device (through the middle), **b)** – shows an illustration of the polarities in this cross section; **c) d) e)** shows the magnetic field strength measured in T, 0cm, 0.4cm, 0.8cm from the surface of the magnetic device shown by the black lines and circles correspond with the same points on the cross section and with the graphs.

Figure S3

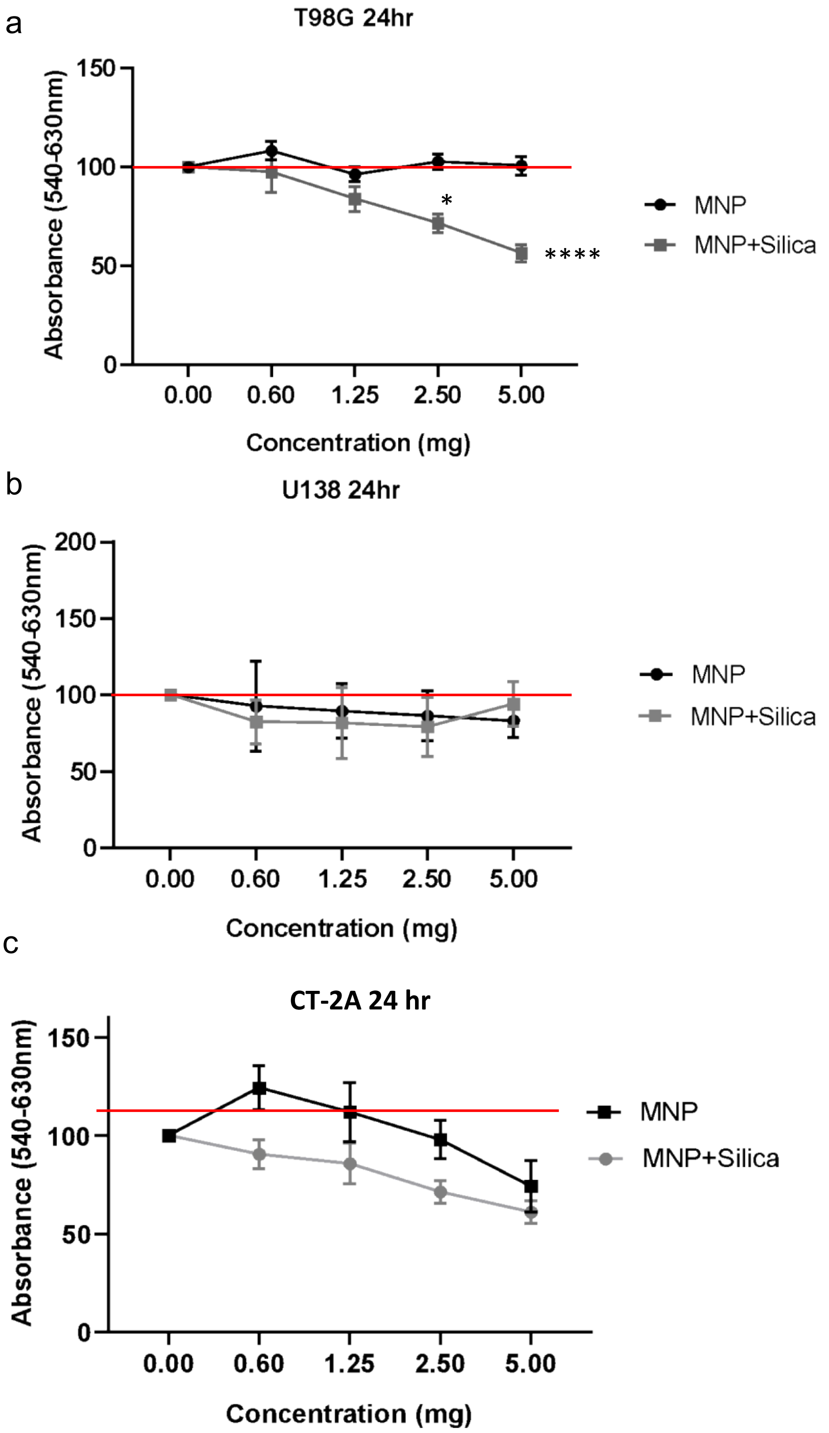

Figure S3: High concentrations of MNPs induce tumour cell death. Human T98G, U138 and murine CT-2A brain tumour cells were incubated with naked MNP (MNP) or silica-coated MNP (MNP + Silica) in a 96 well plate for 24 hours. Alamar Blue assay shows the reduction in cell viability as concentrations of MNP and MNP + Silica increase on all three cell lines; **a)** T98G, **b)** U138, **c)** CT-2A. Data are mean ±SEM of N=6, Independent experiments and were analysed using a one-way ANOVA with Tukey's multiple comparison test, $*p < 0.05$.

Figure S4

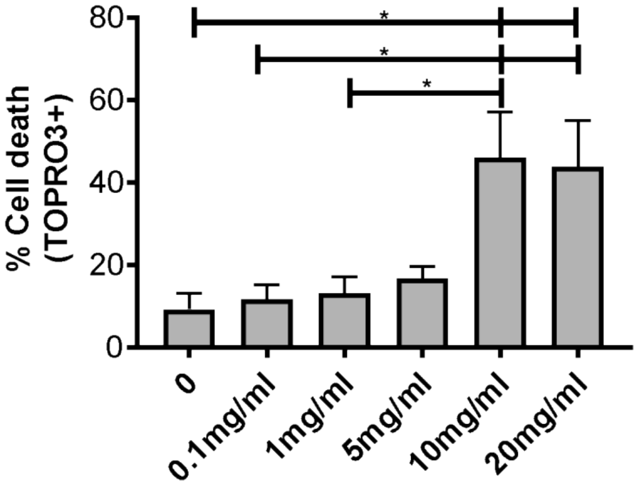

Figure S4: High concentrations of MNP induce PBMC cell death. Human PBMCs were isolated from buffy coats and incubated with different concentrations of iron (0,0.1,1,5,10,20mg/ml) for 24 hours. Cell viability was assessed by flow cytometry using TO-PRO-3 and the amount of cell death was quantified using Flow-Jo software. Data are the mean ± SEM and analysed using a one-way ANOVA with Tukey's multiple comparison test,
$*p < 0.05$.

Figure S5

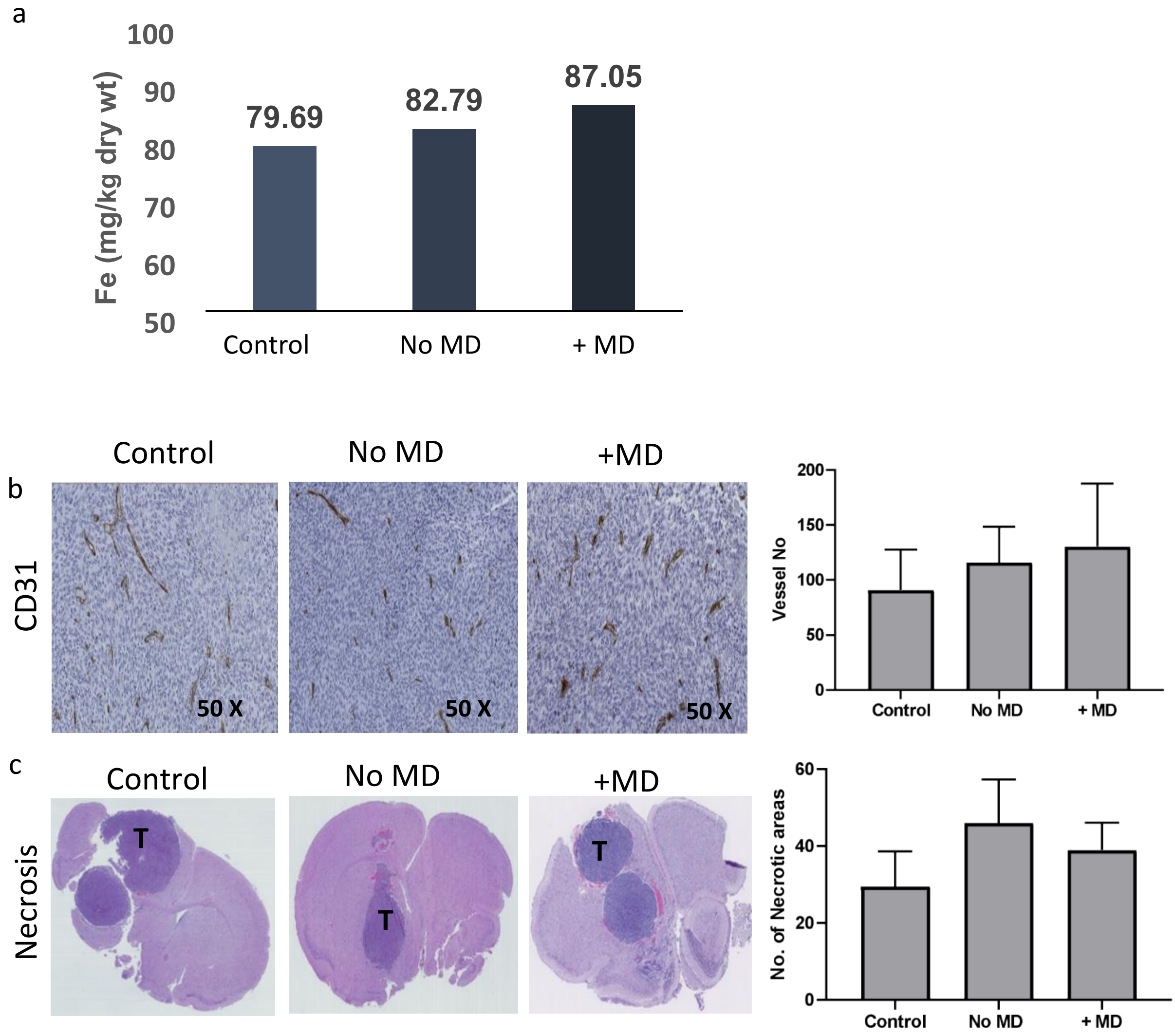

Figure S5: Magnetic guidance of MNP does not affect tumour vascularity or necrosis. Mice underwent intracranial surgery followed by injection of luciferin labelled CT-2A BT cells into the cortical region. Tumour growth was tracked by IVIS imaging. **a)** ICP measurements of iron content (Fe (mg/kg dry wt)) following administration of MNP (5mg/kg) present in digested brain tissue from the three groups in the presence of the magnetic device (+MD) or absence (No MD) and control (no MD) **b)** Sections of the brain (8µm) were stained with CD31 to assess the vessel necrosis following treatment. Arrows indicate CD31 positive staining of the vessels **c)** H & E to determine necrosis within the tumour following treatment. Data was quantified using the Cell Counter tool from ImageJ (Fiji) [National Institutes of Health (NIH), Bethesda, MD, USA] and 5 randomly selected fields of view were imaged for whole brain. Data are the mean and SD for N=3 mice per group. A one-way ANOVA with Tukey’s multiple comparison test was conducted for both stains, $p > 0.05$.

T –Tumour.

Figure S6

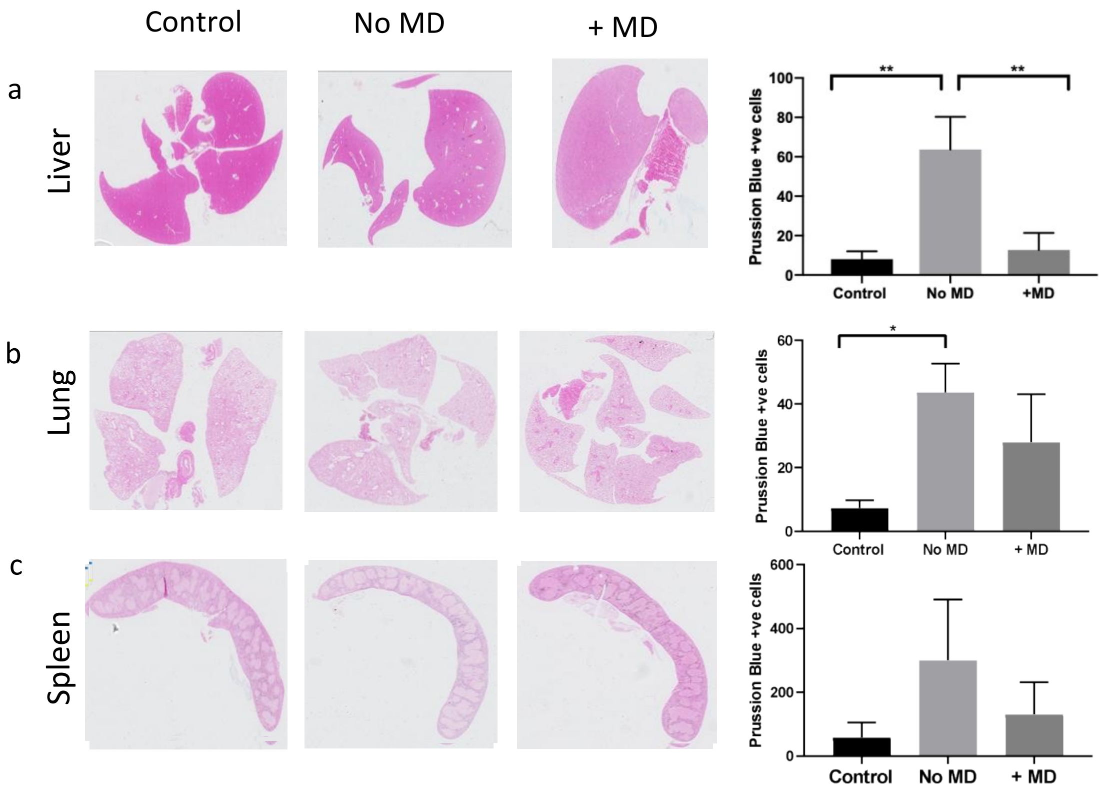

Figure S6: **Biodistribution of MNPs approx.** 2hrs mins after intravenous administration. Mice underwent intracranial surgery followed by injection of luciferin labelled CT-2A brain tumour cells into the cortical region. On day 7 post – surgery mice were administered with MNP (5mg/kg) in the presence of the MD or absence of the MD. Representative Prussian Blue staining of the **a)** Liver, **b)** Lung and **c)** Spleen sections (8µm) and corresponding graphs show the presence of the MNP in these organs. Data was quantified using the Cell Counter tool from ImageJ (Fiji) [National Institutes of Health (NIH), Bethesda, MD, USA] and 10 randomly selected fields of view were imaged per section, Data are the mean SD for N=3 mice per group. A one-way ANOVA with Tukey's multiple comparison test was conducted, p>0.05.

Figure S7

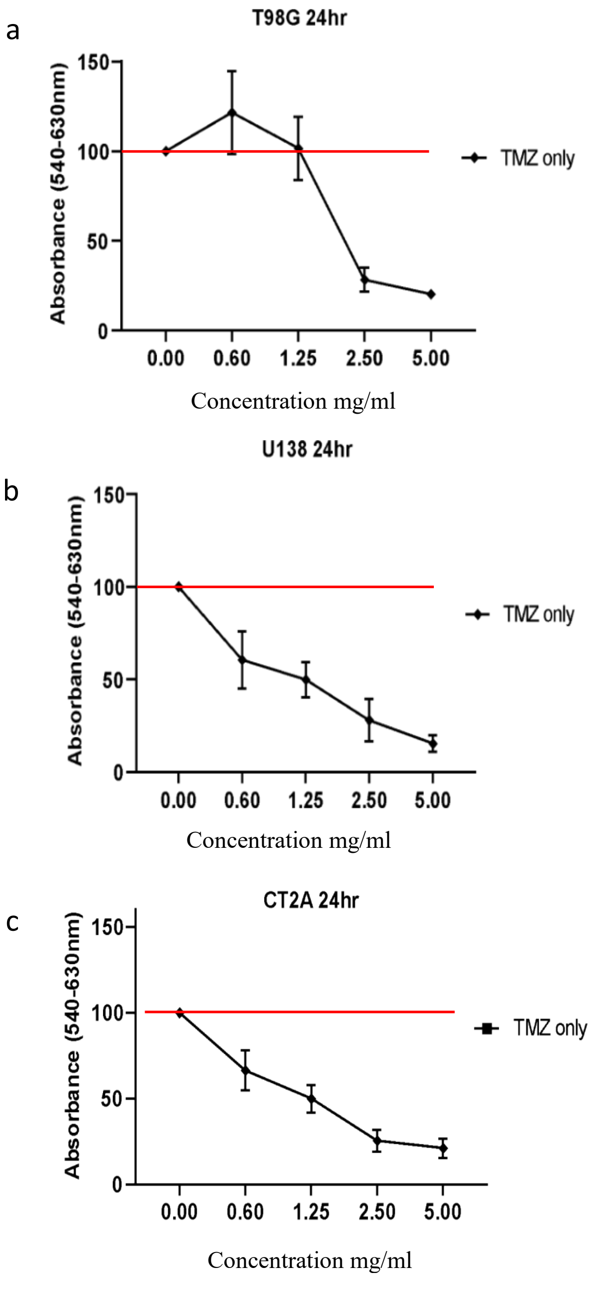

Figure S7: TMZ effectively kill human and mouse BT cells. 5,000 cells were seeded onto 96 well plates in triplicate and incubated with TMZ (5mg, 2.5mg, 1.25mg, 0.60mg) for 24 hours followed by the addition of Alamar Blue (10% v/v) for 4 hours. Fluorescence was read at excitation wavelength 570nm and emission wavelength 585nm using a plate reader **a)** Human cell line (U138) **b**) Human cell line (T98G) **c**) Murine mouse cell line (CT-2A). Data are mean ±SEM and analysed using a one-way ANOVA with Tukey's multiple comparison test compared to vehicle treated cells,

*$p < 0.05$.

Figure S8

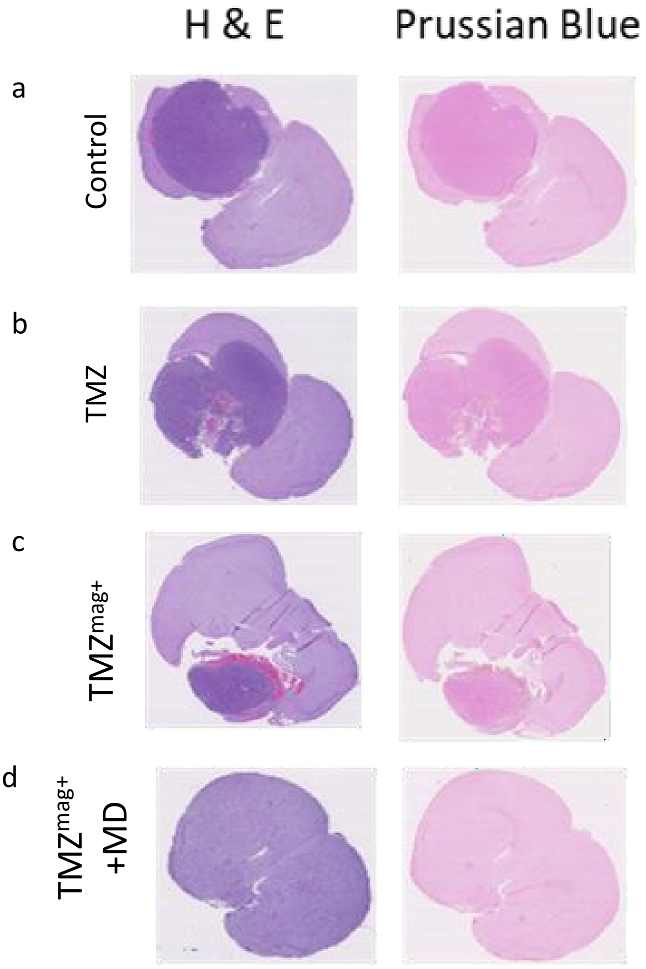

Figure S8: MNP are eliminated from the brain over the experimental period. Mice received 3 doses of i.v. injection after intracranial implantation of luciferin labelled CT-2A brain tumour cells into the cortical region. **a)** 100µl PBS (control), **b)** TMZ only, **c)** TMZ^{mag+} only, **d)** TMZ^{mag+} with the presence of the MD in day 7. **a-d)** representative histology of H&E and Prussian Blue stained brain sections revealed that MNP were no longer present in the brain sections at the end of the experiment. Days post-surgery: **a)** Day 18 (Control), **b)** Day 24 (TMZ only), **c)** Day 24 (TMZ^{mag+}), **d)** Day 48 (TMZ^{mag+} + MD). N=4 mice/group and data was quantified using the Cell Counter tool from ImageJ (Fiji) [National Institutes of Health (NIH), Bethesda, MD, USA] and the whole section was scanned for stained positive cells.

Figure S9

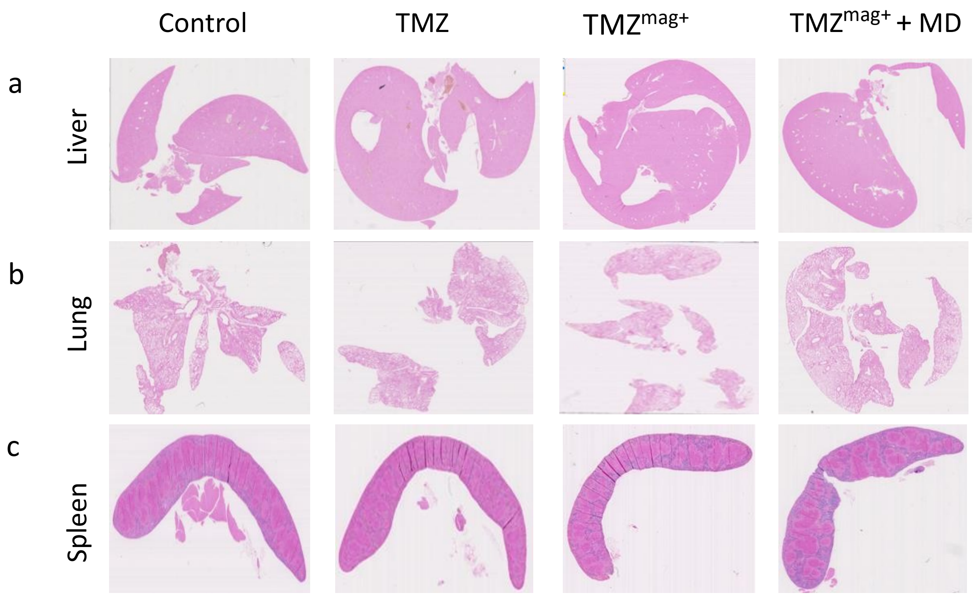

Figure S9: MNP are eliminated from the brain from the Liver, Lung and Spleen. Mice received 3 doses of intravenous injection of: 100µl PBS (control), TMZ only, TMZ^{mag+} only and TMZ^{mag+} with the presence of the MD on day 7. Representative images of Prussian blue staining within the Liver, Lungs and Spleens of mice following treatment days post-surgery – **a)** Day 18 (Control), **b)** Day 24 (TMZ only), **c)** Day 24 (TMZ^{mag+}), **d)** Day 48 (TMZ^{mag+} + MD). N=4 mice/group and data was quantified using the Cell Counter tool from ImageJ (Fiji) [National Institutes of Health (NIH), Bethesda, MD, USA] and the whole section was scanned for positive cells.

Figure S10

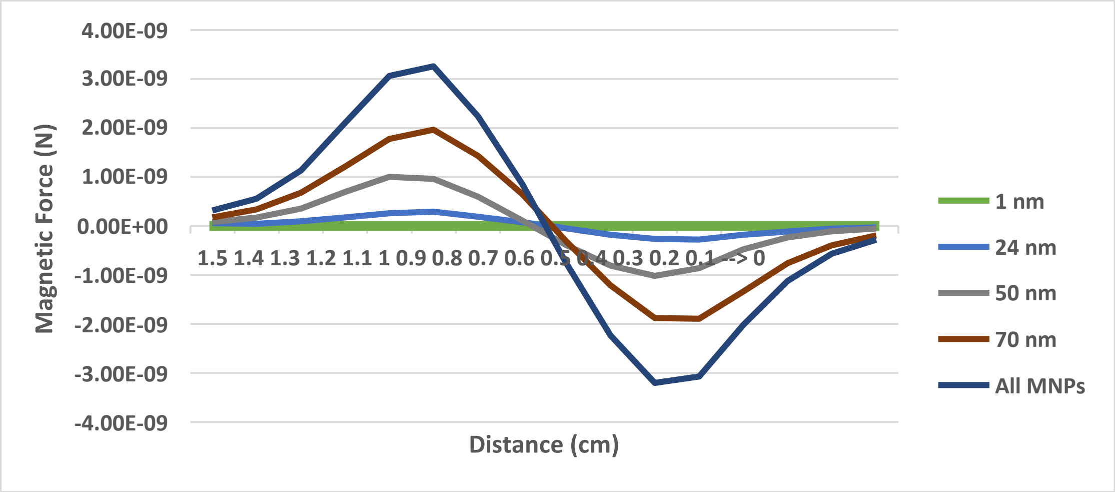

Figure S10. **Calculated magnetic force for different size MNPs vs. Distance.** Green line represents the movement of 1nm MNP, light blue line represents the movement of 24nm MNP, grey line represents the movement of 50nm MNP, brown line represents the movement of 70nm MNP, and dark blue line represents group simulation. This graph shows an increased magnetic force is required with an increase size of MNPs.

Figure S11

a

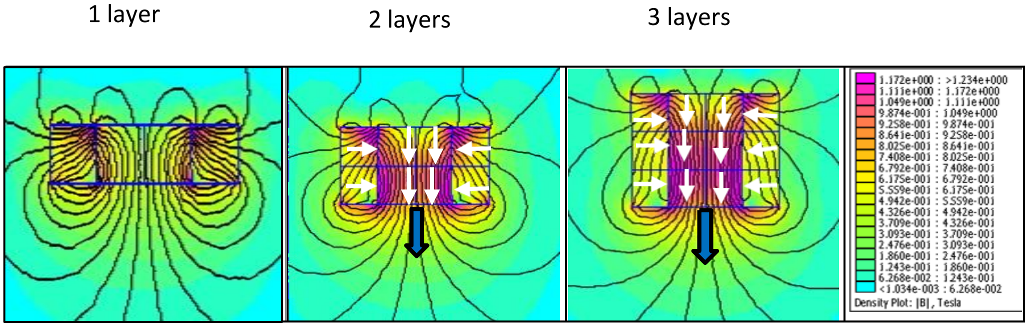

b

Fully assembled magnetic device (FEMM) 3 layers	
Magnetisation orientation	180°,90°,90°,0°
Individual magnets	48
Magnetic strength (B)	1.3 T
Magnetic gradient (G)	13 T/m
Magnetic force (F)	16.9 T^2/m

c

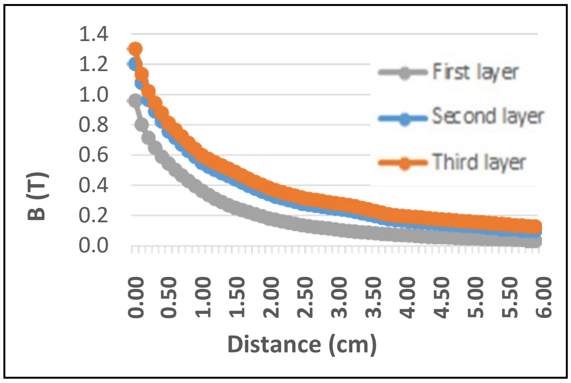

d

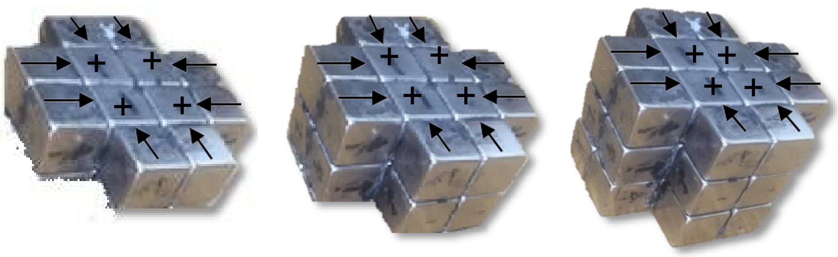

e

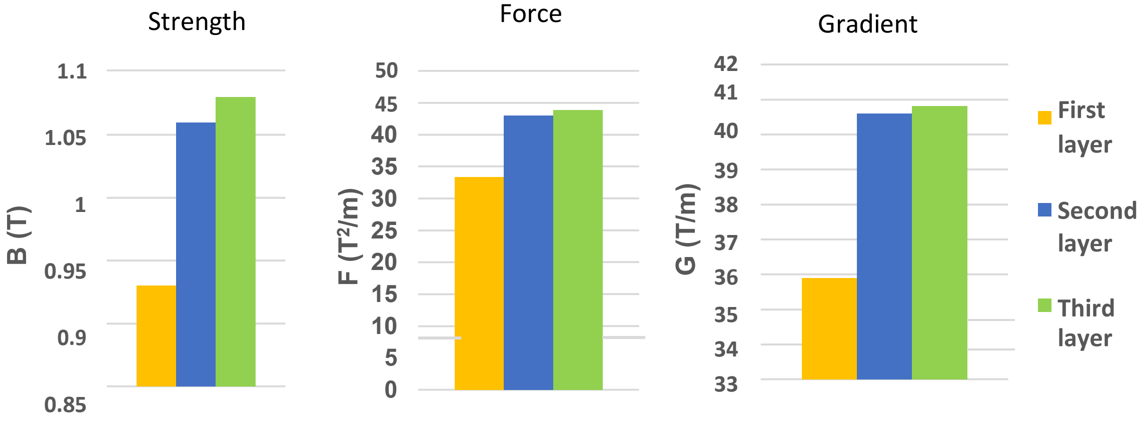

Figure S11: Halbach arrays for the scaled up magnetic device. a) FEMM maps of the first, second and third layer of the magnet respectively, the white arrows display the magnetisation direction (pointing to north). **b)** The magnetic properties of 3 layers, orientations, amount of magnets used and magnetic field strength, gradient and force measured using FEMM at a distance of 5 cm. **c)** The maximum magnetic field strength B(T) of multiple layers measured in FEMM revealed that increasing the layers of magnets produced the highest magnetic field at distances up to 5cm. **d)** Black crosses placed on centre four magnets on the surface of the device refers to the magnetic field strength measurements taken using a Gaussmeter, each magnet element is a volume of 1cm^3 and a single (+) on the magnets refers to magnetization vector set at θ=90°, the black arrows show the

magnetization vector set of θ=180◦ which are orthogonally magnetized to the centre. **e)** Measurements taken show the average magnetic field strength (B), magnetic force (F) and field gradient (G) of each layer

Figure S12

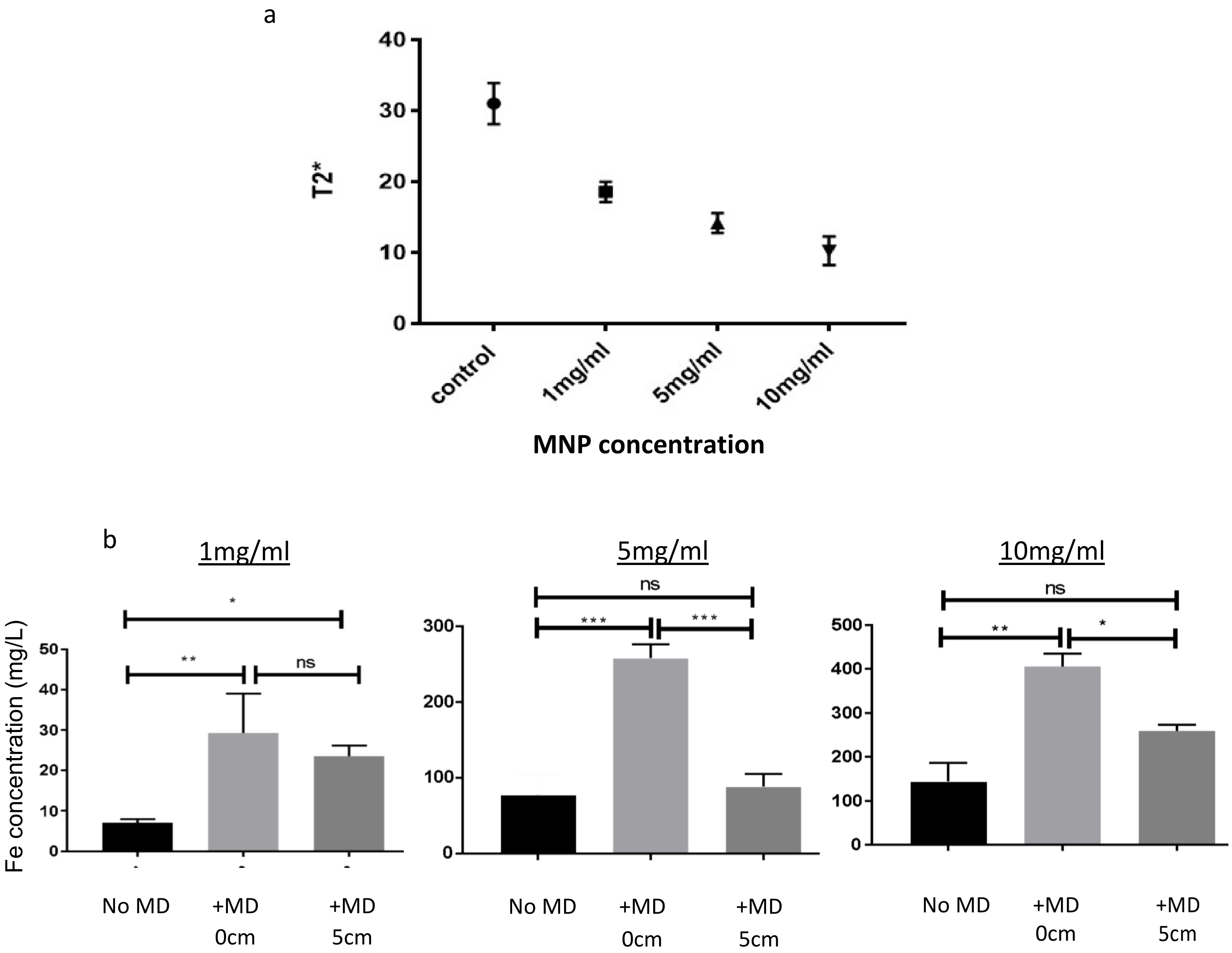

Figure S12. **Scaled up MD targets MNPs at distance of 5cm**. MNP concentration a T2* decreases with increasing MNP concentrations. T2* values were calculated after signal to noise ratios were measured from 3T MRI scans at two different TE values; 4.60s and 20s. The natural logarithm of the signal was calculated and plotted against time. **b** ICP data confirms 'targeting' of MNPs (1,5,10mg/l) that were flushed out of the tumour phantom model. The tumours were located at 0 cm and 5 cm away from the magnet. Data are the mean and SD of N=3 experiments. All data was analysed using one way anova-prism.*p <0.01, **p =0.001 and ***p <0.001.
